# Mapping the research trends of third language acquisition: A bibliometric analysis based on Scopus

**DOI:** 10.3389/fpsyg.2022.1021517

**Published:** 2022-11-03

**Authors:** Zhao Liu

**Affiliations:** School of Foreign Languages, East China Normal University, Shanghai, China

**Keywords:** L3 acquisition, performance analysis, collaboration analysis, thematic map, systematic literature review

## Abstract

L3 acquisition is gaining prominence in the academic community. The cardinal aim of this study is to conduct a bibliometric analysis of research on L3 acquisition. 425 documents from the Scopus database were analyzed with Bibliometrix. To gain a general and systematic overview of research on L3 acquisition, we grounded our study on three main levels of bibliometric analysis: performance analysis, collaboration analysis, and thematic map. By doing so, we identified the most influential sources, authors, affiliations, countries, and documents, the scientific network among different constituents, as well as the evolution of research trends. The results suggest that L3 acquisition has experienced three periods: initial phase (1984–2008), development phase (2009–2014), and burst phase (2015–2022). The results also indicate that: (1) The *International Journal of Multilingualism* is the most steady source contributing to this field. (2) Relevant contributors for each period are recognized, including established and emerging researchers. (3) European countries such as Spain, United Kingdom, Norway, Sweden, and Poland are at the forefront of publication. (4) Collaboration increases over time but is becoming increasingly oriented in European and Anglophone countries. (5) The research hotspots have shifted throughout time, encompass a broad range of fields, and continue to grow. The study results provide insights into the evolving trends of L3 acquisition studies and data to assist researchers in identifying research gaps.

## Introduction

Society nowadays is immersed in a multi-linguistic context, and multilingualism is a common practice for many speakers around the world (De Angelis, 2007; de Bot, 2015). The increase in multilingualism worldwide can be attributed to a number of factors. English remains the *lingua franca*, the language of communication *par excellence*, and is also widely used in other non-English speaking countries as a primary vehicle of communication (Cenoz and Jessner 2000: viii). Social mobility and the rise of immigration resulted in more multilingual speakers. Some countries also have more than one official language (i.e., Spain); hence, a considerable portion of the world’s population speaks multiple languages daily or is learning languages beyond the second language (Cenoz and Genesee, 1998; De Angelis, 2007; Garcia Mayo, 2012; Cenoz, 2013). Another possible motivator is the pragmatic need to assist language planners in determining when to begin third-language instruction in a school setting (Cenoz and Hoffmann, 2003).

Since its inception in the 20th century, third language acquisition (L3 acquisition) has become an essential aspect of research in several disciplines, including linguistics, education, and psychology. However, L3 acquisition has traditionally been categorized as part of second language acquisition, and scholars that investigate third language acquisition tend to focus their study on theories or models for second language acquisition. Not until much later did researchers begin to view L3 acquisition as a separate and unique field of research (Leung, 2007), enriched by multiple subfields across cultural and linguistic boundaries and providing theoretical and empirical data. Following the trend of L3 acquisition, an increasing number of studies on L3 acquisition have been conducted, leading to the necessity to understand its primary status and track its evolutionary path.

Bibliometric techniques serve to gain insight into the field’s structure, social networks, research trends, and themes. So far, bibliometrics has been colored by a wide range of metrics, from calculating publication or citation to combining the trinity of publication, citation, and indexation by using different algorithms (Harzing, 2019). Some systematic literature reviews using bibliometric techniques have already been carried out within the field of linguistics, for instance, on second language acquisition (Zhang, 2020) and bilingualism/multilingualism (Lin and Lei, 2020). However, what is seemly missing is an overreaching bibliometric analysis portraying the research state of L3 acquisition since third language acquisition is not merely an extension of second language acquisition or bilingualism. To this end, this present paper aims to apply the bibliometric techniques implemented on Bibliometrix (Aria and Cuccurullo, 2017) to delve into the current research state of L3 acquisition, generate knowledge maps, and conduct a systematic and comprehensive analysis of the existing literature.

The remainder of the paper is structured as follows: Section 2 overviews the scope definition and major bibliometric studies in this field. The methodology is documented in Section 3, including a detailed description of data, processing, and tools. Section 4 demonstrates the results of the analysis. Section 5 concludes with the discussion and conclusion and outlines the limitations of this study.

## Background

### Scope definition

Despite the growing interest, L3, or the third language, is still a nebulous concept with a wide range of definitions. It is defined by Cenoz (2003, p. 29) as “the acquisition of a non-native language by learners who have previously or are acquiring two languages.” According to De Angelis (2007), the term “third language acquisition” seems inappropriate because the word ‘third’ lays great emphasis on the third language to the exclusion of the speaker’s previous dominant languages. Therefore, she is in favor of “third or additional language acquisition,” which refers to all languages acquired after L2 without prioritizing preference to any. Thus, the so-called L3 does not imply that this language was learned after the first two. As noted by Herdina and Jessner (2000, p. 85), the learning of a third language results in the use of three languages, two languages other than the mother tongue can be acquired simultaneously or consecutively (Cenoz, 2003, p. 29). In a similar spirit, Hammarberg (2001) argues that it is inadequate to classify a language as a third language based on the order of acquisition. In contrast to L2, which a person acquires after L1, he defines L3 acquisition as the language now being learned. Wunder (2011) described the L3/Ln “as an umbrella term for any non-native language including and learnt beyond the chronologically third foreign language.” Other terms in the literature include “multiple language acquisition,” “multilingual acquisition,” and “third or additional language acquisition” (see De Angelis (2007) for a thorough examination of the terms). L3 can be acquired in a variety of circumstances, and hence, the terminology L3 encompasses a large number of subcategories. For instance, Hoffmann (2001) differentiates five kinds of trilinguals based on the conditions and the setting in which they acquire their trilingualism: (1) Children raised with two different home languages and the community language. (2) Children raised in a bilingual community who also speak a different home language. (3) Bilingual learners who learn a third language at school. (4) Bilinguals who acquire a third language due to immigration purposes. (5) Individuals in a trilingual society. Given the purpose of this study, we contend that the two most prevalent terms in the literature, “L3 acquisition” and “third language acquisition,” are equally suitable for our investigation.

### Bibliometric analysis

The present study is founded on the methodological foundation of bibliometrics. Even though the term “bibliométrie” was first used in 1934 by Paul Otlet in *Traité de Documentation* (Rousseau, 2014) and was not anglicized by Pritchard (1969, p. 349) until 1969, its concept dates decades earlier (Osareh, 1996; Wallin, 2005; Roemer and Borchardt, 2015). Cattell (1906) probably made one of the earliest bibliometric attempts when he analyzed the distribution of 1,000 American scientists across disciplines, organizations, and regions. According to Pritchard (1969, p. 349), bibliometrics is the application of mathematical and statistical methods to books and other media of communication to gauge the distribution structure, quantitative relationship, and quantitative management of literature and information. That is to say, by taking a mathematical and statistical stance, it classifies data and builds up representative summaries (Broadus, 1987) to provide a quantitative approach to the literature reflected in bibliographies (White and McCain, 1989, p. 119).

Evolving around the trinity of publication, indexation, and citation (Nylander et al., 2020), bibliometrics can be used to measure and estimate the evolution of a certain subject, as well as classify and describe the characteristics of literature (Guler et al., 2016; Bornmann and Marewski, 2019). In addition, it is beneficial to decipher developing trends in a field, detect collaboration patterns, and investigate the intellectual structure of a certain topic in the existing literature (Donthu et al., 2021, 2022). Consequently, well-executed bibliometric studies can lay the groundwork for advancing a field in novel and significant ways; they enable and empower scholars to (1) obtain a comprehensive overview, (2) identify research gaps, (3) generate novel research ideas, and (4) position their intended contributions to the discipline (Donthu et al., 2021).

Given its advantages in capturing the state of the art of a given field in a fast and efficient fashion, especially in dealing with large datasets (Ramos-Rodríguez and Ruíz-Navarro, 2004) in an objective fashion (Diodato, 1994), bibliometrics is widely used in the scientific field to describe patterns of publication within a given field or body of literature (Begeny et al., 2018; Modak et al., 2019), both in social sciences and natural sciences. In light of the advantages of bibliometric techniques, the purpose of the current study is to apply this technique to examine the rapidly expanding field of L3 acquisition and to depict its current research status.

### Studies on linguistics using bibliometric analysis

Compared to other studies in natural science, the proliferation of bibliometrics research in language studies is relatively nascent. It has only been 5 years since Liao and Lei (2017) claimed that bibliometric studies on linguistics are in short supply. Recently, numerous bibliometric studies have been published within this field: applied linguistics (de Bot, 2015; Liao and Lei, 2017; Gong et al., 2018; Chen et al., 2019; Lei and Liu, 2019a), translation studies (van Doorslaer and Gambier, 2015; Zanettin et al., 2015; Ping, 2021; Rovira-Esteva et al., 2021), education (Hung and Zhang, 2012; Hernández et al., 2017; Gong et al., 2018), bilingualism and multilingualism (Lin and Lei, 2020; Gutiérrez and Duque, 2021), discourse analysis (Swales, 1986; White, 2004; Huan and Guan, 2020; Xiao and Li, 2021; Wang et al., 2022), L2 pronunciation research (Demir and Kartal, 2022), L2 writing (Arik and Arik, 2017; Sun and Lan, 2021; Swatek et al., 2022), language teaching and learning (Jiang et al., 2020; Chen et al., 2021; Hiver et al., 2021; Hyland and Jiang, 2021; Bakelak and Reyes, 2022), among others.

Apart from focusing on a specific research topic, the bibliometric analysis in the linguistic field can also manifest at the journal level. For instance, scholars have undertaken such a study by examing the publications of a determined journal: *Applied Linguistics* and *Journal of Linguistic* (Ezema and Asogwa, 2013), *System* (Lei and Liu, 2019b), *German Journal of Writing Centres* (Bromley et al., 2020), *Teaching English as a Second Language Electronic Journal* (Pearson, 2022), *Review of Applied Linguistics in Language Teaching* (Zhong and Liu, 2022), *Language Testing* (Dong et al., 2022), *Language Problems and Language Planning* (Li and Liu, 2013), and *Journal of Language and Linguistic Studies* (Syahi̇d and Qodi̇r, 2021).

Studies are also conducted at the level of region or country. Barrot (2017) undertook a study to determine the research influence and productivity of Southeast Asian countries in the field of language and linguistics. Lei and Liao (2017) present the results of a bibliometric analysis of the evolution of linguistic research in China from 2003 to 2012. The objective of Mohsen (2021) was the research contributions of the Kingdom of Saudi Arabia to the field of applied linguistics that were indexed on the Web of Science from 2011 to 2020.

All previous bibliometric studies have demonstrated the effectiveness of bibliometric analysis on linguistics-related subjects, focusing on a specific topic, journal, or region. However, no previous studies have employed this analyzing technique in third language acquisition. Two studies, among all others, are highly pertinent to the present investigation, the one by Zhang (2020) on second language acquisition and the one by Lin and Lei (2020) on bi−/multilingualism. Zhang (2020) examined studies on second language acquisition using data retrieved from the Web of Science and mapped the field’s evolving trajectories. The study collected research published in 16 international journals between 1997 and 2018, and the author identified the most-cited papers, journals, research institutions and regions, and authors, as well as the shift in research interests. According to the results, SLA has witnessed a boost in the number of publications. In addition, while the majority of studies address SLA from the cognitive approach, other approaches such as sociocultural theory and complex theory start to emerge. Moreover, some research topics have gained popularity over time (e.g., translanguaging, teacher cognition, multilingualism), and a number of topics are becoming less popular (e.g., focus on form, extrinsic motivation, topic familiarity, and phonological awareness). Additionally, the author identified the most influential publication sources, classified into two groups (SLA/SLT and psycholinguistic/bilingualism), as well as regions that contribute the most to SLA (North America). Lin and Lei (2020) examined bilingualism and multilingualism research in Linguistics and Education over the past two decades (2000–2019) and discovered that most studies adopt either a psycholinguistics/cognitive or a teaching/learning perspective. Moreover, a set of hot themes (e.g., emergent bilinguals, metalinguistic awareness, phonological awareness, executive control) and cold themes (e.g., vocabulary knowledge and bilingual students) are identified, as well as the top journals based on the number of publications and the most cited documents. An interesting finding is that scholars seem to be more interested in adopting a multilingual perspective than the traditional bilingual perspective.

While both studies have revealed some research trends in language acquisition and bi−/multilingualism, contributing to the existing area of foreign language acquisition, their findings leave room for L3 acquisition, which is the focus of the present study. Though it stems from second language acquisition, third language acquisition presents differences with both second language acquisition and bilingualism. Multiple studies (Cenoz, 2000, 2001, 2003; Gallardo del Puerto, 2007) have already suggested that third language acquisition is more complex than second language acquisition. For instance, linguistic knowledge, and foreign language learning experience, which cognitively contribute to a higher degree of both language awareness and metalinguistic knowledge, as well as better communicative skills, differentiate L3 learners from L2 learners (Bardel and Sánchez, 2020). Hence, the learning process for L2 and L3 learners also tend to differ (Sánchez, 2020) since, at the onset of foreign language acquisition, L3 learners already possess certain kinds of linguistic advantages (experience, knowledge, and learning strategies) that L2 learners are lack of (Gibson and Hufeisen, 2003; Jessner, 2006). In addition, potential interactions between the language systems in the mind of the multilingual speaker and their access to universal grammar are among the key differences in the learning process (Falk and Bardel, 2010).

The search terms used in Lin and Lei (2020) were *bilingualism* and *multilingualism*, and the results contain studies on both lines of research. However, the interchangeable use of these terms may result in a common misconception in academia (Sánchez, 2020). Multilingualism is a broader and more complex term than bilingualism, considering that it may encompass multiple language contacts and diverse learning settings. Instead of including all publications indexed in the database WoS on SLA, Zhang (2020) has selected the 16 most influential journals on SLA without performing a term search to exclude papers irrelevant to SLA published in these sources. Moreover, “current models of SLA and bilingualism cannot adequately explain the unique traits that form the character of TLA” (Marx and Hufeisen, 2004, p. 142) since the development, process, the affecting factors, among other factors, present such a uniqueness that we can not equal third language acquisition to second language acquisition. In this sense, the results of the two studies may not fully account for third language acquisition. Hence, while previous research by Lin and Lei (2020) and Zhang (2020) has contributed greatly to understanding the state-of-art foreign language acquisition, a more comprehensive and concrete analysis of studies on L3 acquisition, including both performance analysis and scientific mapping, appears to be lacking.

### Aim of the present study

As mentioned previously, scholars have conducted an extensive study on bibliometric studies related to linguistics or a branch of linguistics and achieved outstanding results, demonstrating the significant potential for future research. Despite the popularity of L3 research, our knowledge regarding its general development over the last few decades has so far remained somewhat limited, as no previous study has performed a bibliometric analysis in this field. As such, we cannot evaluate its impact or judge the areas where it exerts the most effect, the kind of knowledge that is particularly needed considering its interdisciplinary nature. In reality, bibliometric methods provide new perspectives on the knowledge status and trends in a certain field. Therefore, this research undertakes a bibliometric approach to map the development of L3 acquisition. More specifically, this study aims to answer the following four research questions:

- What is the chronological development of the L3 acquisition from 1984 to 2022?
- Who/Which are the most influential authors, sources, institutions, countries, and documents contributing to L3 acquisition?
- Can any observation be made on the interaction or collaboration among different research constituents (authors, institutions, and countries)?
- Is there any change in terms of research themes?

## Materials and methods

**Figure 1:**
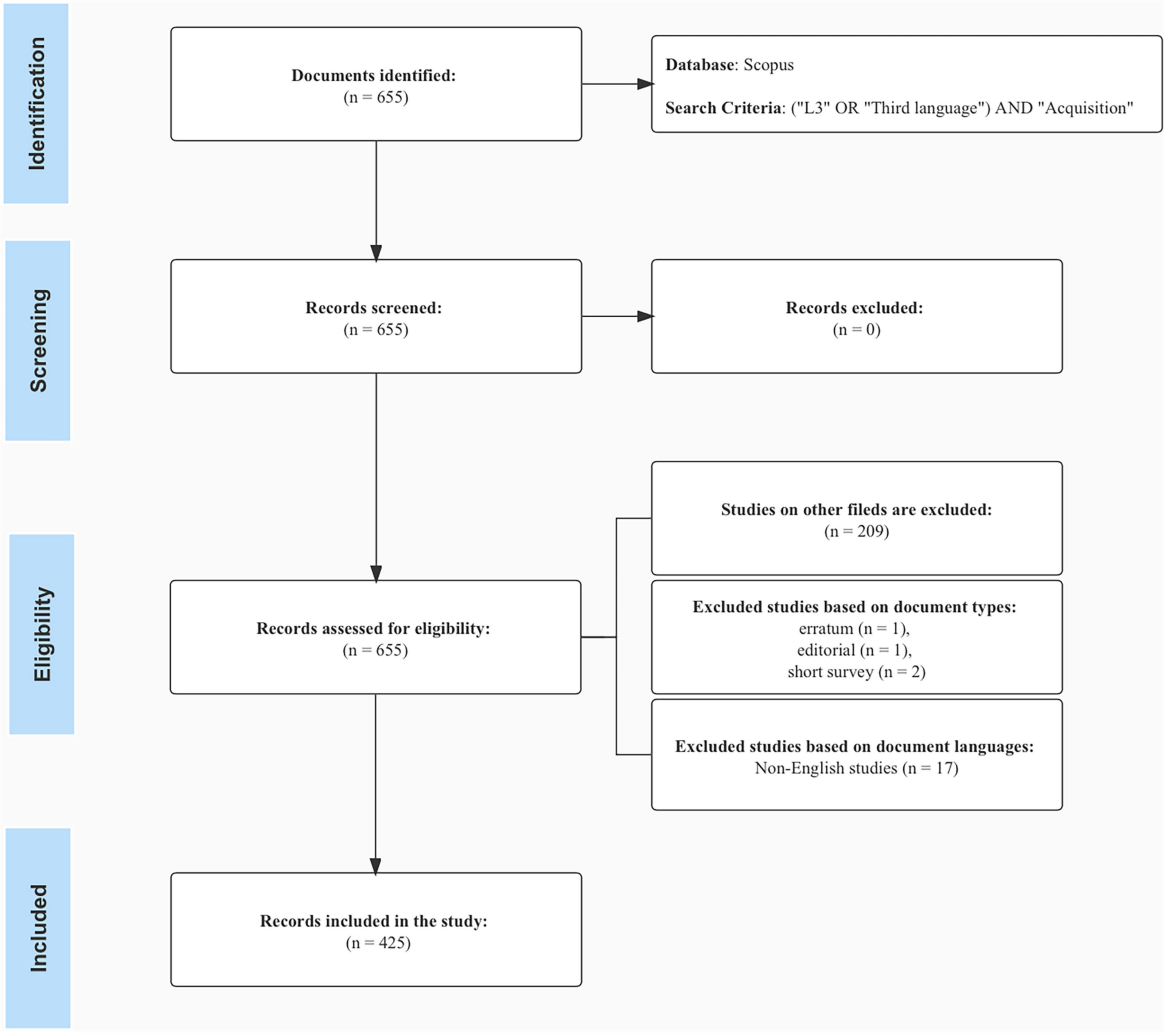
Data collection flow chart, adapted from Aria and Cuccurullo (2017) and Firdaus et al. (2019).

This section is aimed at supplying details of this bibliometric analysis. It gives further detail on the corpus construction and data analysis so as to ensure the consistency, replicability, and transparency of this study (Gusenbauer and Haddaway, 2020). The PRISMA guidelines (Page et al., 2021) were utilized to construct the reporting pool through a sequential three-stage process (identification, screening, and inclusion). The macrosteps for conducting this research are presented in Figure 1.

### Corpus construction

#### Search strategy

Several well-known bibliometric databases have been widely employed to conduct the bibliometric study: Web of Science, Scopus, and Google Scholar, among others (Chadegani et al., 2013; Fernandez-Llimos, 2018; Martín-Martín et al., 2018; Roldan-Valadez et al., 2019).

Even though much previous research has indicated WoS database as with higher quality and broader coverage in terms of documents (Liu et al., 2015; Elaish et al., 2019; Birkle et al., 2020), the present study used the core collection database of Scopus as the literature source for data retrieval. In order to select the proper database for the present study, we adhered to the following criteria. First, a title search with the same syntax was carried out on WoS and Scopus. The search yielded 491 documents on the WoS and 655 on Scopus. Hence, Scopus seems to contain more documents than WoS for the specific search topic of this study. Secondly, the Bibliometrix tool was used for data analysis in the present study. As suggested by the developers of Bibliometrix, it is not suitable to merge datasets from these two databases, as they use a different approach to codify the bibliographic metadata. Moreover, Scopus is recommended to work with Arts and Humanities studies. In contrast to WoS and Scopus, Google Scholar has a broader range of indexing, including unpublished documents among its citation sources (Roemer and Borchardt, 2015). However, even though Google Scholar has more comprehensive coverage of sources for citations, it does not differentiate between academic and non-academic citations, which could lead to bias (De Bellis, 2009) and the discriminating standards that the other two databases maintain (Roemer and Borchardt, 2015).

The literature search was conducted in July 2022, with the initial search producing 655 documents with the stated keywords across disciplines. The principle of collecting data is as follows. First, a search with keywords was conducted on Scopus. The search range was the title, keywords, and abstract. The leading search terms were “third language” and “acquisition.” Both terms were related by the Boolean operator “AND.” Within the category, we used the Boolean operator “OR,” which indicates that either term satisfies the condition. The general search categories in this study were: “l3” OR “third language” AND “acquisition.” In this stage, we did not limit the time span in order to gather the evolution over time since the first appearance of studies on L3 acquisition.

#### Inclusion criteria

To guarantee that the retrieved articles were relevant to the research topic, we followed several steps to ensure that the data met the inclusion criteria. First, we read their abstracts carefully and manually sifted unrelated studies. For instance, during the data search, research articles on medicine, chemistry, and biology were retrieved, but they were irrelevant to the research direction of this paper. The rationale for not applying the filter in the first stage is as follows: several studies on L3 acquisition also take a psychological perspective; if a language-only filter is applied, we fear losing potentially relevant studies for our research. Therefore, we manually reviewed the abstracts of the documents to sift out irrelevant research.

After manual inspection, the search was refined by document type and language (see Supplementary Material). We first excluded the erratum, short surveys, and one editorial document, yielding 442 documents. Note that book chapters are decided to be included in the research pool after a thorough revision for since these studies pertain to relevant works dedicated to third language acquisition (i.e., *Third Language Acquisition and Universal Grammar*). In addition to English, Spanish, German, and French appear to be the most common languages in which studies are published. Given the limitation in analyzing techniques, the search was limited to English language results (or at least English is one of the publishing languages).

The final sample comprises 425 documents retrieved as research objects from the Scopus database (see Supplementary Material). The documents in the dataset were published from 1984 to June 2022. The sources are somewhat diverse, with 157 different sources. On average, the document we incorporated in this study is 6.68 years old. The average citation per document is 16.36. In terms of authorship, we find 605 authors. There is an average of 1.43 authors per document; less than half of the sources are contributed by only one author (188 documents, 44.24%). About 77.8% of the authors are engaged in collaboration (Authors of non-single-authored docs / all authors), and the international co-authorship is 17.88%. The co-authors per document are 2.04.

#### Data cleaning

Before performing all the analyses, we cleaned the data manually and created thesaurus files, and embedded them within the Bibliometrix interface. Thus, we were able to avoid coding errors and combine variants of an author’s name or an institution’s name with different spellings into a single unit. The first phase of data cleaning involves manually correcting author names. For example, “Jennifer Cabrelli Amaro” and “Jennifer Cabrelli” refer to the same author; therefore, these variations were recoded into “Jennifer Cabrelli.” The second round of cleaning entails correcting the names of the affiliations. Occasionally, the institution’s name is given in its native language. For example, “University of the Basque Country” and “Universidad del País Vasco” (Spanish) refer to the same institution; therefore, English names were selected to avoid confusion. The third stage involves combining the author’s keywords that convey the same subject. For example, “third language” and “L3” refer to the same concept and were therefore recorded as the same keyword. However, items with only a moderate similarity were not combined to allow for a greater variety of reader interpretations, i.e., “crosslinguistic transfer” and “transfer.”

### Data analysis

#### Data analysis tool: Bibliometrix

Previous studies have proven the Bibliometrix’s effectiveness in conducting bibliometric studies (Firdaus et al., 2019; Linnenluecke et al., 2020; Pahrudin et al., 2022). Bibliometrix is a unique R-tool for comprehensive scientific map analysis, developed using statistical computation and graphic R languages following a logical bibliometric workflow, including all significant bibliometric analysis methods. It is used to evaluate a specific subject’s quantity and development trend (Hao et al., 2018). As a valuable assessment of books, articles, and other publications in a particular field over time, Bibliometrix can also help researchers observe the performance of various subjects.

In this study, the data was analyzed using Bibliometrix (Aria and Cuccurullo, 2017) and self-written scripts in RStudio (RStudio Team, 2022). The final sample retrieved from the Scopus database was exported in BibTex format, and the data was imported into Bibliometrix and converted into an R data frame. In the data analysis process, manual and semi-manual calculations were performed when necessary to enhance research validity.

#### Data analysis metrics: Performance analysis and science mapping

In bibliometrics, performance analysis and science mapping are two prevalent techniques. Performance analysis, descriptive in nature, can aid those interested in a particular topic in comprehending its fundamental concepts and present level of development. Not only can it indicate the evolution of a specific topic of study, but it can also identify core papers and journals. Science mapping is centered on the knowledge structure, allowing for the visualization and networking of the intricate relationships between different constituents. As scholars (Noyons et al., 1999) suggested, performance analysis and science mapping should be implemented in bibliometric analysis. Hence, in this present study, we incorporate both quantification of performance and knowledge mapping technology. The techniques for bibliometric analysis manifest across three primary levels of analysis in this study: (1) performance analysis, (2) collaboration analysis, and (3) thematic map. The first focuses on accounting for the contribution of research constituents, while the latter two probe into the dynamic development of the research field.

The performance analysis evaluates the contributions of research constituents (document, author, institution, country) to a specific field, elucidates sample features, and analyzes its primary performances by measuring the research field (e.g., number of publications, number of citations), identifying the most significant items (e.g., most cited, most prolific), and determining the impact of their activity (Ramos-Rodríguez and Ruíz-Navarro, 2004; Cobo et al., 2011; Fusco et al., 2020).

Both collaboration analysis and thematic map belong to science mapping, defined as “a spatial representation of how disciplines, fields, specialties, and individual papers or authors are related to one another” (Cobo et al., 2011, p. 147). It examines the relationships between research constituents (Ramos-Rodríguez and Ruíz-Navarro, 2004; Cobo et al., 2011) and constructs the bibliometric network.

Studies of scientific collaboration (Newman, 2001; Yan and Ding, 2012) were conducted to identify the most significant relationships between the different research components, commonly used in studies to determine the social structure of a field. This is achieved through a social network analysis in which the nodes are the authors, their institution, or the country to which the institution belongs. The edges (links) are constructed based on the nodes that co-authored an article, which not only can shed light on the relative importance of research constituents but also enrich the bibliometric evaluation (Andrikopoulos and Economou, 2016; Cisneros et al., 2018; Andersen, 2021; Baker et al., 2021).

The thematic map also pertains to the science mapping aspect. In this study, we performed a theme analysis, inspired by Cobo et al. (2011) and developed by Aria and Cuccurullo (2017), to gain insight into the field’s evolution. Bibliometrix uses the thematic map to describe the conceptual structure of the topic, which consists of a word co-occurrence network analysis to discover the center of inquiry in a scientific field and its prominent themes and trends.

## Results

The bibliometric analysis commenced with the description of the general bibliometric statistics. Then, this section is organized around findings regarding the aforementioned research questions: (1) the most influential sources (number of contributions, Bradford’s Law, h-index), authors (Lotka’s Law for authorship pattern, author’s contribution), affiliations, and countries (number of contributions), and documents (number of citations), (2) collaboration among scholars, countries, and institutions; and (3) thematic map based on the author’s keyword. Given space limitations, detailed information and data for analysis will be found in Supplementary Material.

### Performance analysis: Sample characteristics

#### Publishing trends

**Figure 2:**
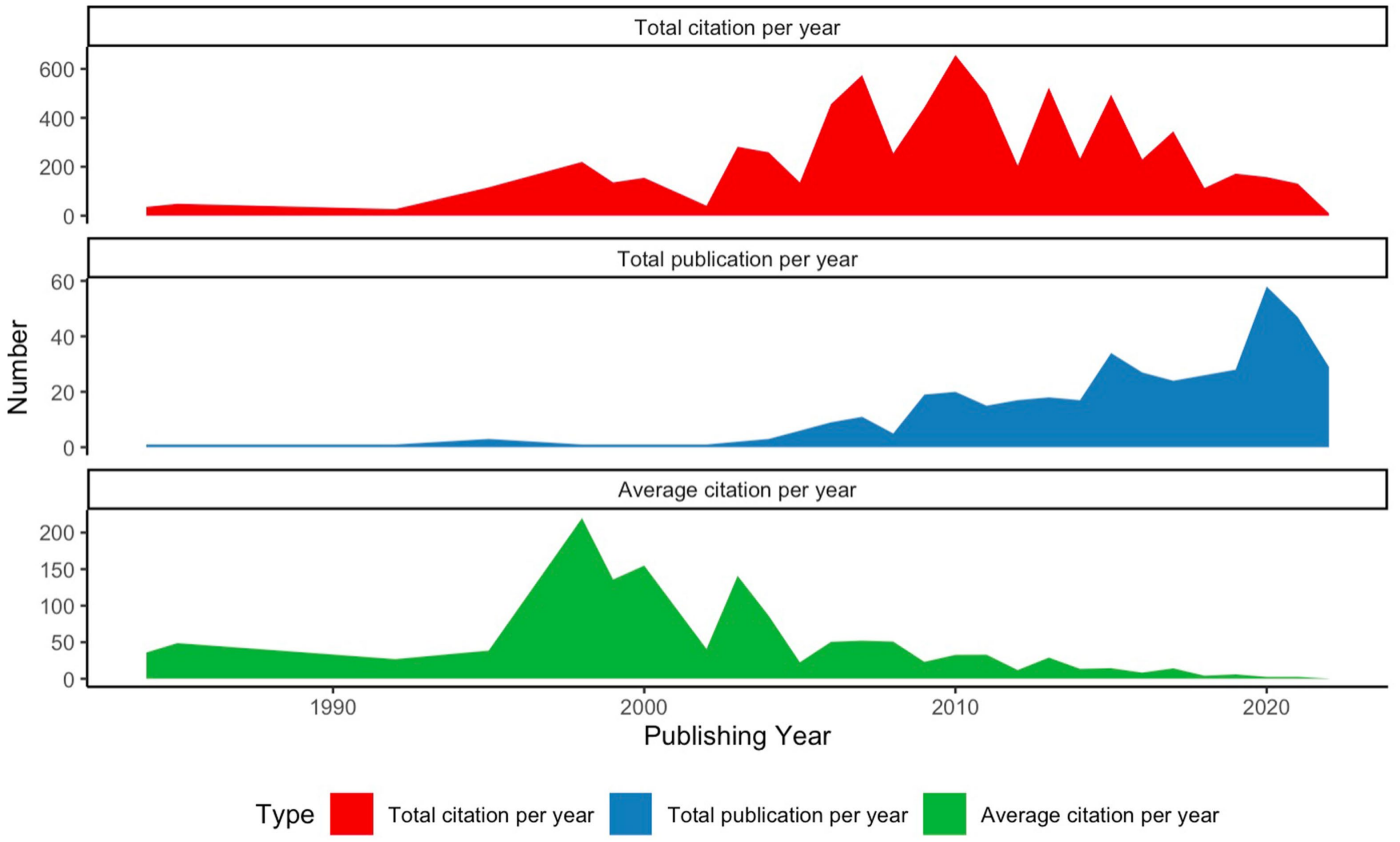
Publication (total number of publications) and citation (total citation and average citation per document per year) trends in L3 acquisition from 1984 to 2022.

The chronological development of publication can aid comprehension of different developmental stages (Guo et al., 2021). As evidenced in Figure 2, the research on L3 acquisitions shows an increasing trend, from 1984 to 2022, especially after 2009. Moreover, several peaks can be observed from the figure, for instance, 2007 (*N* = 11), 2009 (*N* = 19), 2015 (*N* = 25) and 2020 (*N* = 58), where an abrupt increase can be found. Even though a decline can be found from 2021 to 2022, it should be noted that at the time of data collection, July 2022, the data for 2022 was still incomplete, and some publications have not been included in the database yet. The highest number of articles was published in 2020, with 58 documents or 13.65 percent of the 425 documents published in the study period. This is closely followed by the publication in 2015 with 25 documents (5.88%).

Previous studies have divided the period according to calendar decades (Lei and Liu, 2019a,b) or observations made by scholars (Dong et al., 2022). However, following Demir and Kartal (2022), we employed the non-parametric Mann-Kendall test and Pettitt’s test for change-point detection to identify a potential change point.

The Mann-Kendall test showed a significant difference at 0.05 level for the time span between 1984 and 2022: (z = 5.9813, *p* = 2.214e-09, S = 301.00, tau = 0.821687). According to the results of Pettitt’s test, there was a possible change point in 2008. Then, we also conducted a non-parametric Mann-Kendall test for the time range 2009 to 2022, and a trend was also observed (z = 2.7962, *p* = 0.005171, S = 52.00, tau = 0.5745944). Pettitt’s test for single change-point detection pointed to 2014 as a possible changing period. Another non-parametric Mann-Kendall test was conducted for 2015 to 2022, and no significant difference was found (z = 0.90113, *p* = 0.3575, S = 7.00, tau = 0.3333333).

Hence, the publication in this field can be roughly divided into three phases, with 2008 and 2014 as cutting edges. The first period stretches from 1984 to 2008, during which the annual publication volume seems relatively slow, demonstrating that this is still an understudied topic, which can be seen as the infancy or initial stage of L3 acquisition. However, since 2009, the volume of publications started to grow; therefore, the second phrase is defined as the steady development stage of L3 acquisition in this study. Since 2015, the number of research papers has greatly increased, signaling the beginning of the third period, which we refer to as the burst development stage.

Detailed information regarding the data structure across the three time periods is given in Table 1. Generally, an increasing trend can be observed in the number of sources, documents, author’s keywords, authors, author’s appearances, authors of single-authored documents, single-authored documents, co-authors per document, and the international co-authorship rate.

**Table 1:** Main information about the collection reported separately for the three periods.

	Period	Period 1	Pediod 2	Period 3
Main information	Timespan	1984: 2008	2009: 2014	2015: 2022
	Sources	27	46	112
	Documents	46	106	273
	Average citations per doc	59.63	24.13	6.059
	Average citations per year per doc	3.162	2.08	1.267
	Author’s keywords	85	284	727
Authors	Authors	63	171	428
	Author appearances	76	205	587
	Authors of single-authored docs	21	48	79
Authors collaboration	Single-authored docs	28	55	105
	Documents per author	0.73	0.624	0.638
	Co-Authors per Doc	1.65	1.93	2.15
	International co-authorships %	6.522	15.09	20.88

However, the average citation per document and the average per year per document seem to show a negative variation. Documents published in the first period have the most citations per document, with 59.63, followed by sources published in the second period, with 24.13 per document. Documents published in the last period have the fewest citations per document, at 6.059. The tendency toward a higher citation rate for articles published in previous issues is reasonable, as the earlier an article is published, the greater the likelihood that it will be cited (Tahamtan et al., 2016; Aksnes et al., 2019).

In our study, we also observe a decrease in the rate of sole authorship (single-authored documents / total number of documents): in the first period (60.87%), the second period (51.89%), and the third period (38.46%). Our findings differed slightly from previous studies (Ezema and Asogwa, 2013; Barrot, 2017; Syahi̇d and Qodi̇r, 2021), who reported a low degree of collaboration in the field of linguistics and suggested that sole authorship might be the norm in the fields of language and linguistics due in part to the solitary and competitive nature of the two fields, which is exacerbated by a relative lack of collaborative agendas (Barrot, 2017). For instance, 63.2% of the whole documents in their study are single-authored (Ezema and Asogwa, 2013). Ezema and Asogwa showed that there are very few research collaborations among Southeast Asian countries in the field of language and linguistics, and Syahïd and Qodïr (Syahi̇d and Qodi̇r, 2021) reported that more than half of the data was authored by sole authors. Despite the fact that the rate of joint authorship in our study may be lower than in other science studies [i.e., 76.64% for safety culture in van Nunen et al. (2018)], our analysis indicates an increase in the rate of collaboration over time.

#### Source

The impact of different sources is explored *via* the following parameters: number of publications, Bradford’s Law for identifying the core journals, and h-index for source impact.

The documents in the data were published in 157 sources in total. The top-10 sources based on the number of publications represent nearly half of the documents retrieved for this study (46.83%). Consistent with findings in other research domains, a small number of prolific sources contributed significantly to the publication of L3 acquisition. According to Scopus categories, all are peer-reviewed journals and classified primarily within the Linguistic and Language field. The most abundant source, the *International Journal of Multilingualism*, represents approximately 14.82% of the total number of documents retrieved (63 documents), followed by *Second Language Research* (6.82%) and the *International Journal of Bilingualism* (4.94%).

We compared these results with that of previous studies by Zhang (2020) on L2 acquisition and by Lin and Lei (2020) on bilingualism and multilingualism. Only two journals in our study (*Second Language Research* and *Bilingualism: Language and Cognition*) was included in Zhang’s study of the top 16 SLA journals, and five journals in our study were included in Lin and Lei’s bibliometric study on bi−/multilingualism. However, the most contributed journal in our study, the *International Journal of Multilingualism*, is not among the top 20 journals in the field of bilingualism/multilingualism studies by Lin and Lei.

With regard to the evolution across the three periods, in the first period, very few studies are reported in our data. Detailed revision of the most productive sources revealed that the most stable source is the *International Journal of Multilingualism*, which has consistently ranked first throughout the three periods. The second most influential journal in all three periods is *Second Language Research* (3rd in the first and second periods, with a rise in the third period). Sources like *Journal of Multilingual and Multicultural Development* and *Second Language Learning and Teaching* dropped out of the list, and more journals related to bilingualism *(International Journal of Bilingualism*, *Bilingualism* and *Linguistic Approaches to Bilingualism*) entered the list during the last period.

In the second period, we identified a book as the main source, i.e., *Third Language Acquisition and Universal Grammar*, a special volume in the Series of Second Language Acquisition published in 2009. The nine papers in this collection take a generative linguistic approach to investigate UG-related topics. As one of the early monographs devoted to L3 acquisition, this collection has reported empirical data on a variety of languages. According to the editors, the collection is designed to spark interest, debate, and an intellectual interchange between scholars in the study of L3 acquisition Leung, (2009: xiii).

The contribution of sources to the field of L3 acquisition has also been evaluated using Bradford’s law, which describes how documents in the data are spread between journals. Bradford’s law states that “if scientific journals are arranged in order of decreasing productivity of articles on a given subject, they may be divided into a nucleus of periodicals more particularly devoted to the subject and several other groups of zones containing the same number of articles as the nucleus” (Patra et al., 2006, p. 29). It is therefore useful for identifying the core journals within a field.

We conducted an analysis per period, and, in our data, the first zone of the nucleus comprises three journals in the first period: *International Journal of Multilingualism*, *Second Language Research*, and *Journal of Multilingual and Multicultural Development*. However, in the second period, the latter dropped out from the core sources, wherein the former two remained to be most contributing to the field. In the third period, recall that there are also more sources during this stage; besides the *International Journal of Multilingualism* and *Second Language Research*, which have always remained on the list, we also identified four new journals: *International Journal of Bilingualism*, *Bilingualism*, *Linguistic Approaches to Bilingualism*, and *International Journal of Bilingual Education* and *Bilingualism*. This finding is also consistent with our previous analysis of the most relevant sources, namely that these five journals are the most prolific in the field of L3 acquisition.

Another measurement used for evaluating the source impact is the Hirsch index (H-index), an author or a journal’s number of published articles, each of which has been cited in other papers at least h time (Aria and Cuccurullo, 2017). Generally speaking, the higher the h-index, the more influential a source is. The *International Journal of Multilingualism* is the journal with the highest impact across the three periods. We also identified the *Journal of Multilingual and Multicultural Development* and *Second Language Research* as the sources with the highest h-index in the first period. In the second period, while the latter remains one of the most influential sources, the former was replaced by the *International Review of Applied Linguistics in Language Teaching*. In the third period, the scenario changed slightly; the *International Journal of Multilingualism* remained at the top, and the *International Journal of Bilingualism* and *Bilingualism* appeared as the most influential source. There seems to overlap between the most influential and productive journals.

#### Authors

The research questions also concern the most prominent authors. Here we use Lotka’s Law to assess the most-dominant authorship, the number of publications to identify the most prolific authors, and the author’s active publishing timeline to monitor the trend.

Lotka’s Law describes the frequency of publishing by authors on a particular topic. The number of authors publishing a certain number of articles is a fixed ratio to the number of authors publishing a single article. As the number of articles published increases, the frequency of publication decreases (Aria and Cuccurullo, 2017), 79.9% of the authors have written only one article, and more than 91.1% of authors have published fewer than two documents; therefore, they are considered occasional scholars in this field. A similar exploration has been conducted for different periods, and it seems that no core authors were identified for the first and the second periods. However, in the third period, more authors are stable in scientific productivity in this field.

**Figure 3:**
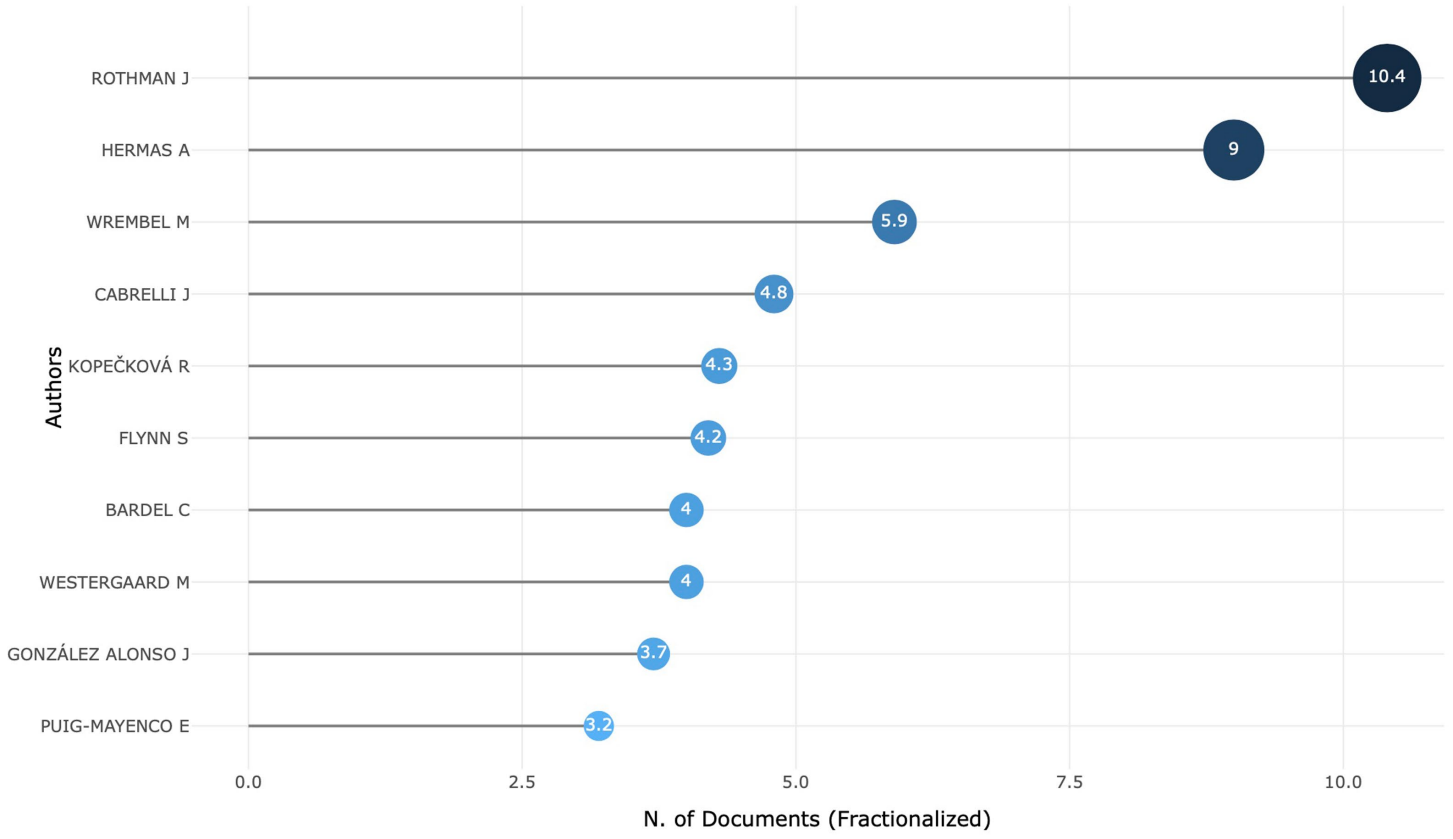
Top 10 ranking authors with the highest number of publications.

In order to identify the most productive authors, we present the fractionalized number of documents for the top 10 authors (Figure 3). Some contributions have more than one author; hence, the number of documents we present here is fractionalized, assuming equal contribution among all co-authors. As Figure 3 shows, Jason Rothman is identified as the most relevant author by the number of documents, followed by Abdelkader Hermas. Other relevant authors include Magdalena Wrembel, Jennifer Cabrelli, Romana Kopečková, Suzanne Flynn, Camilla Bardel, Marit Westergaard, Jorge González Alonso, and Eloi Puig-Mayenco.

To gauge the possible difference across periods, we conducted a similar analysis for each period. Indeed, different names were identified throughout the period, demonstrating that the field of L3 acquisition was still undergoing consolidation. In the first period, Jasone Cenoz, Ulrike Jessner, and Yan-kit Ingrid Leung are the most contributing authors. In the second period, scholars like Camilla Bardel and Jean-Marc Dewaele kept working in this field. Also, more scholars were devoted to L3 acquisition, such as Jason Rothman, Suzanne Flynn, and Magdalena Wrembel. In the third period, researchers such as Jason Rothman, Magdalena Wrembel, and Jennifer Cabrelli continue to work in the field, also coming at the top area of the rank list are names of other scholars such as Abdelkader Hermas, Marit Westergaard, Jennifer Cabrelli, Romana Kopečková, and Jorge González Alonso. Consequently, the top 10 authors for the whole timespan (1984–2022) mirror that in the third period.

**Figure 4:**
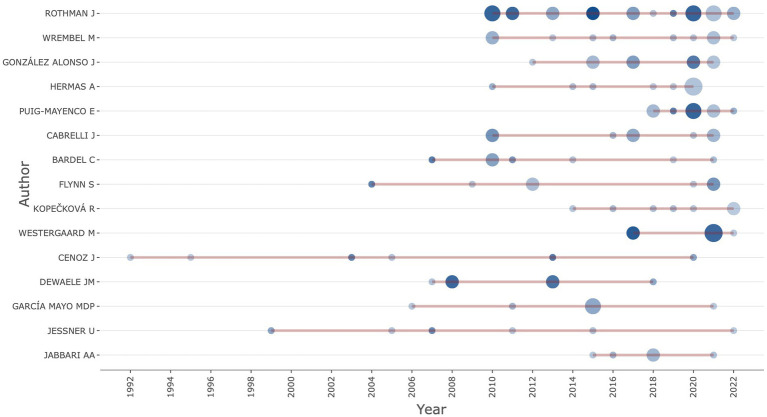
Authors’ Production over Time (top 15).

In order to gain a complete view of the evolution of the authors, we plot the author’s production over the time investigated in the study (Figure 4). The line represents an author’s timeline in terms of publication. The bubble size is proportional to the number of documents, and the color intensity is proportional to the total citation per year. It is interesting to note that most of these authors started to publish extensively after 2010. Jasone Cenoz has the most extended timeline, from 1992 to 2020, and is the only scholar who has been active in this field for almost three decades. Scholars such as Ulrike Jessner and Suzanne Flynn keep contributing to the field. The recent development of the L3 acquisition has also seen some emerging scholars, such as Eloi Puig-Mayenco, whose active contribution started after 2018.

#### Affiliations and countries

The distribution of affiliations and countries is a way to visualize the distribution of publications geographically. Table 2 reports the 10 most productive affiliations according to the corresponding author’s institution and the total number of publications. Note that some authors report more than one affiliation, and, in this case, the classification was determined by order of precedence, and the first affiliation was taken into account. A total of 336 affiliations were identified in this study. As shown in Table 2, the University of the Basque Country is the most productive affiliation, followed by The Arctic University of Norway, Stockholm University, and Adam Mickiewicz University. In the list, all these countries belong to either European countries or Anglo-Saxon countries.

**Table 2:** Information on the first ten most productive affiliations ordered by the number of total publications: Affiliation, the country it belongs to, and the total number of publications.

Affiliation	Country	Documents
University of the Basque Country	Spain	26
UIT The Arctic University of Norway	Norway	22
Stockholm University	Sweden	15
Adam Mickiewicz University	Poland	11
Université du Québec à Montréal	Canada	9
University of Reading	United Kingdom	9
University of London	United Kingdom	9
Norwegian University of Science and Technology	Norway	8
University of Arizona	United States	8
University of Southampton	United States	8

Additionally, we divided the data into three periods. Examining the total number of contributions by period reveals that the University of the Basque Country (Spain) held a leading and dominant position in the first two decades, with an enormous lead over second place in the first period but a smaller lead in the second. In the first period, institutions primarily belong to European nations. In the second and third periods, a few universities from the United States and Canada appeared on the list. The Arctic University of Norway surpassed the University of the Basque Country in the third period. Overall, it appears that the contribution of institutions has varied considerably over time.

**Figure 5:**
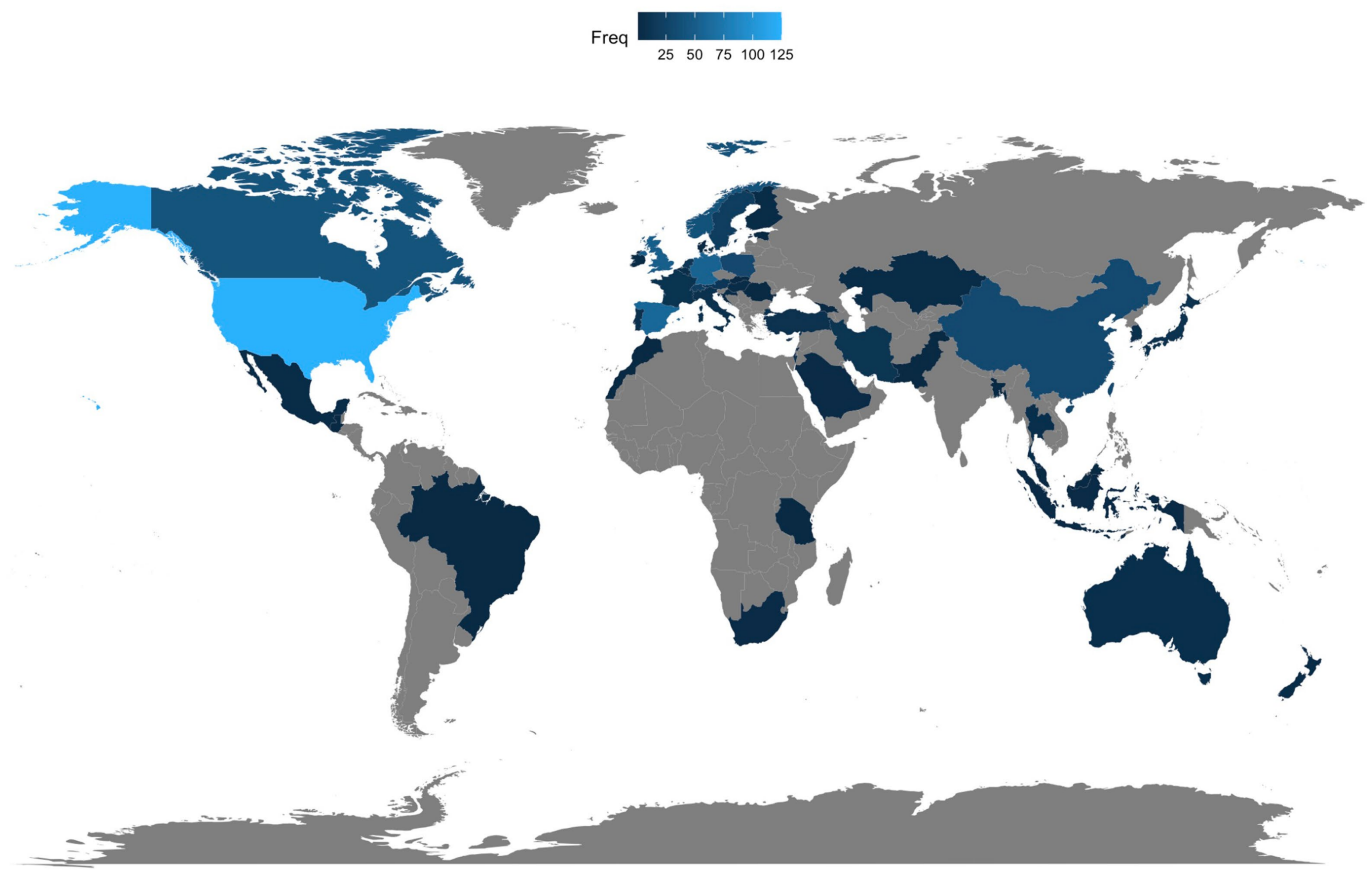
Scientific production by country. Different shades of blue indicate different productivity rates: grey indicates no contribution, and dark blue stands for high productivity. Freq denotes the frequency.

Figure 5 shows the author’s appearances by country and presents a geographical distribution. According to the developers of Bibliometrix, each article will be counted as many times as there are (co)authors. As shown in Figure 5, the geographical distribution of documents is dispersed and distributed across several continents. Nonetheless, the majority of publications are still centered in Anglo-Saxon (e.g., United States, Canada) and European countries (e.g., Spain, United Kingdom, Germany, Norway, Poland). The United States has the most author appearances (125), followed by Spain (60), Germany (56), the United Kingdom (53), Canada (42), Norway (40), and Poland (31). Additionally, we identified several emerging nations, including China (32). One can wonder why the United States occupies first place in Figure 5 despite not being the leading country in the ranking of institutions with the highest contributions in Table 2. After a thorough review of the data, we found that the United States has numerous organizations dedicated to L3 acquisition, contributing to a great sum.

Moreover, we also analyzed the data by period and found that the geographical distribution of contributing countries extended mainly from European and Anglo-Saxon countries (first period) to Asian and African countries (second period) and, in the third period, more countries and continents are involved in conducting related studies.

#### Documents

The most relevant publication is assessed *via* the number of total citations. Table 3 lists the top 10 publications per total citation, the first of which was published by Costa, Santesteban, and Ivanova in 2006 (Costa et al., 2006). In this paper, the authors reported four empirical experiments on the code-switch performance of bilinguals involving different languages they dominate (from L1 to L4). This work stands out as the one with the highest total citation, followed by the paper co-authored by Williams and Hammarberg (1998), Cenoz (2003), Flynn et al. (2004), and Dewaele et al. (2008). The ranking changes slightly if we consider total citations per year. The paper authored by Rothman (2015) is the one with the highest citation per year. Also, he is the only author whose contributions have appeared more than once on this list (Rothman, 2011, 2015), both on the Typological Primacy Model he proposed.

**Table 3:** Top 10 most global cited documents in L3 acquisition from 1984 to 2022.

*N*	Author(s)/Year	Title	TC	TC per year
1	Costa et al. (2006)	How do highly proficient bilinguals control their lexicalization process? Inhibitory and language-specific selection mechanisms are both functional	292	17.18
2	Williams and Hammarberg (1998)	Language switches in L3 production: Implications for a polyglot speaking model	220	8.80
3	Cenoz (2003)	The additive effect of bilingualism on third language acquisition: A review	217	10.85
4	Dewaele et al. (2008)	Effects of trait emotional intelligence and sociobiographical variables on communicative anxiety and foreign language anxiety among adult multilinguals: A Review and empirical investigation	215	14.33
5	Flynn et al. (2004)	The Cumulative-Enhancement Model for language acquisition: Comparing adults’ and children’s patterns of development in first, second and third language acquisition of relative clauses	212	11.16
6	Bardel and Falk (2007)	The role of the second language in third language acquisition: The case of Germanic syntax	192	12.00
7	Rothman (2011)	L3 syntactic transfer selectivity and typological determinacy: The Typological Primacy Model	168	14.00
8	Jessner (2008)	Teaching third languages: Findings, trends and challenges	161	10.06
9	Sanz (2000)	Bilingual education enhances third language acquisition: Evidence from Catalonia	155	6.74
10	Rothman (2015)	Linguistic and cognitive motivations for the Typological Primacy Model (TPM) of third language (L3) transfer: Timing of acquisition and proficiency considered	140	17.50

Year refers to the year the paper is published. TC, total citation.

These documents can be roughly divided into three fields, taking into consideration their research focus: (1) Bilingualism and third (Ln) acquisition, such as the role of L2 on L3 (Williams and Hammarberg, 1998), bilinguals performance in L3/Ln (Costa et al., 2006), and bilinguals’ (dis)advantages on acquiring an L3 (Sanz, 2000; Cenoz, 2003; Bardel and Falk, 2007). (2) Models to account for L3 acquisition: Cumulative Enhancement Model (Flynn et al., 2004), Typological Primacy Model (Rothman, 2011, 2015). (3) Education and multilingualism (Dewaele et al., 2008; Jessner, 2008).

It should not be surprising that a few of the most-cited documents are from the second or third period, as it takes time for an article to accumulate a large number of citations, as suggested by several scholars. For instance, Ezema and Asogwa (2013) found that citations to sources more than 20 years old account for more than 43 percent of their study. We further split the data into three periods to identify the most frequently cited documents throughout each period.

Indeed, there is substantial overlap between the lists of the top 10 most-cited publications for the first period and the overall dataset. We can add only one additional reference to the first list (Klein, 1995). This contribution falls within the first data category we have identified, i.e., bilingualism and L3 acquisition. In academic circles, whether the L2 and L3 learning processes are comparable and the fundamental differences between the two have been extensively debated (de Bot, 2015).

The documents in the second period can be divided into three fields considering the research foci: (1) Bilingualism and multilingualism (Lüdi and Py, 2009; Cenoz, 2013; Dewaele, 2013; Duff, 2013). (2) Syntactic transfer: L2 status factor (Falk and Bardel, 2010, 2011) and Typological Primacy Model (Rothman and Cabrelli Amaro, 2010; Rothman, 2010, 2011). (3) Learners’ performance in multilingual education (Engel de Abreu and Gathercole, 2012).

In the third period, studies on syntax and transfer keep on proliferating (Giancaspro et al., 2015; Rothman, 2015; Slabakova and García Mayo, 2015; González Alonso and Rothman, 2017; Rothman et al., 2019; Puig-Mayenco et al., 2020). Apart from the Typological Primacy Model and the L2 status factor that have already appeared in the second period, we contemplated more models to account for L3 acquisition: the Scalpel Model (Slabakova, 2017) and the Linguistic Proximity Model (Westergaard et al., 2017). We also found a study on learning motivation and the multilingual status of Turkey as a foreign language (Thompson and Erdil-Moody, 2016).

### Collaboration analysis

The collaboration analysis of different research constituents provides insight into the academic publication network formed in this field. This section presents the collaboration analysis among authors, institutions, and countries.

#### Collaboration among authors

To comprehend any long-term auctorial collaboration, the co-authorship network’s minimum edge was adjusted to two to remove one-time collaborations.

**Figure 6:**
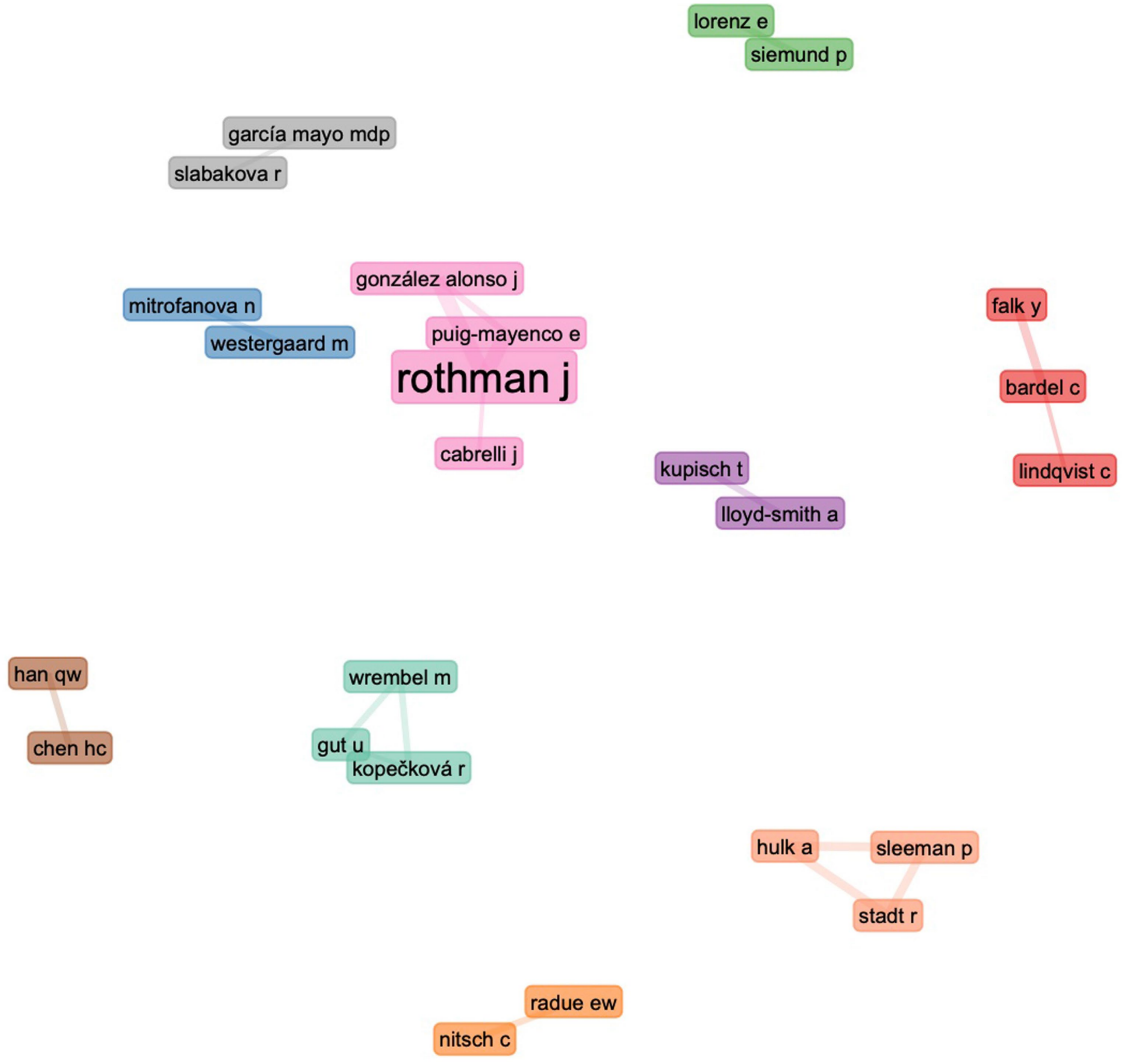
Collaboration between authors.

According to our data, 25 scholars have collaborated with other academics multiple times and are clustered into 10 groups. As seen in Figure 6, the primary scholars maintain a relatively close collaborative relationship. It is also important to note that author collaboration appears to be institution- or country-specific; for instance, researchers from the same university or country tend to collaborate more frequently. The data reveal that collaboration was less widespread during the first period than in the second and third; in the second and third periods, there are more collaborative clusters.

#### Collaboration among institutions

**Figure 7:**
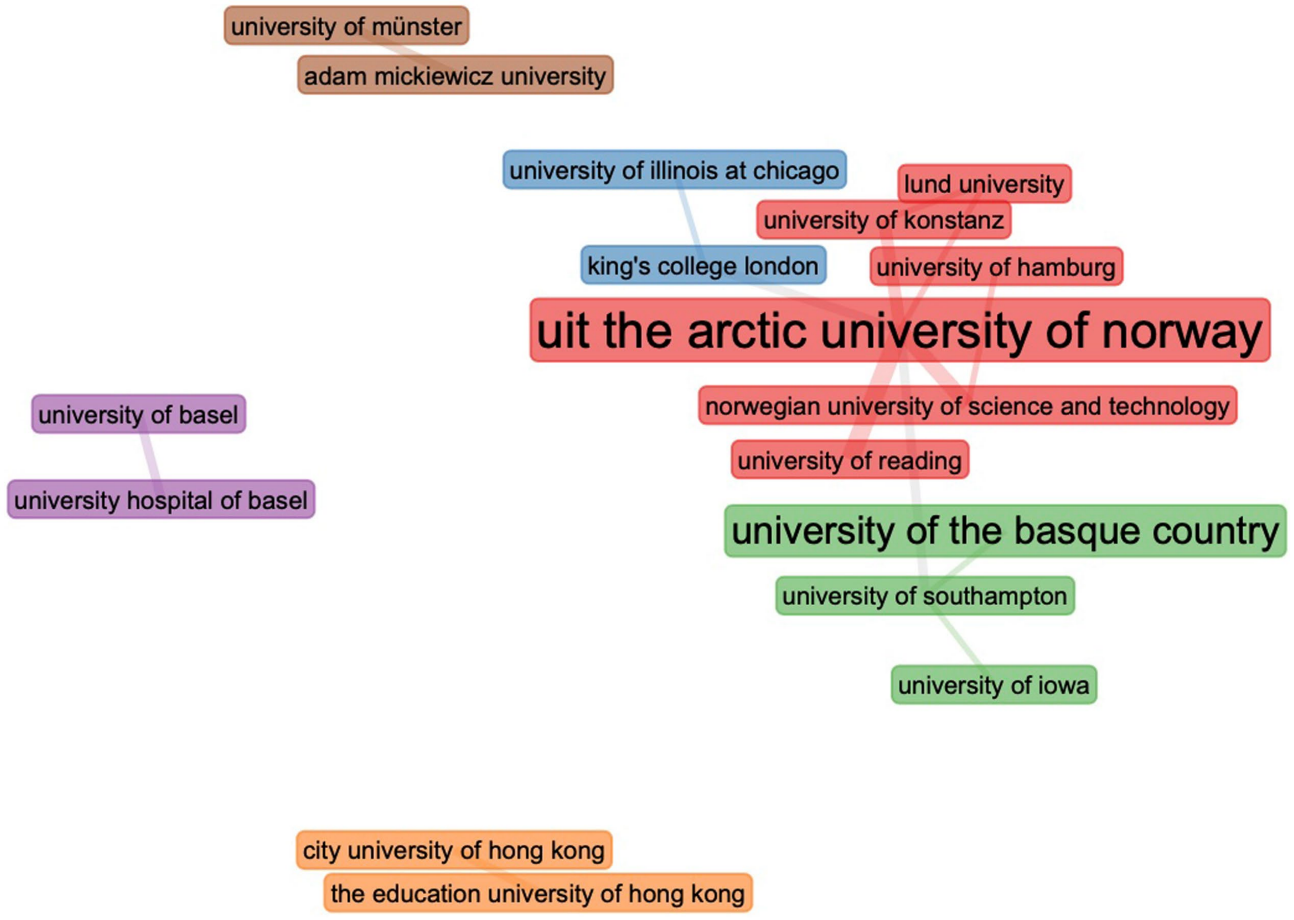
Collaboration between institutions.

Figure 7 depicts the collaboration between academic institutions in six clusters. The largest cluster (in red) comprises institutions from Norway, Sweden, Germany, and the United Kingdom, and the Arctic University of Norway is situated in the cluster’s center. The second cluster (in blue) consists of institutions from the United Kingdom and the United States. The third cluster (in green) consists of Spain, the United Kingdom, and the United States. It is important to note that both the fourth (in brown) and fifth (in orange) clusters are comprised of institutions from the same country; the fourth from Switzerland, and the fifth from China. The last cluster (in purple) consists of German and Polish institutions.

#### Collaboration among countries

**Figure 8:**
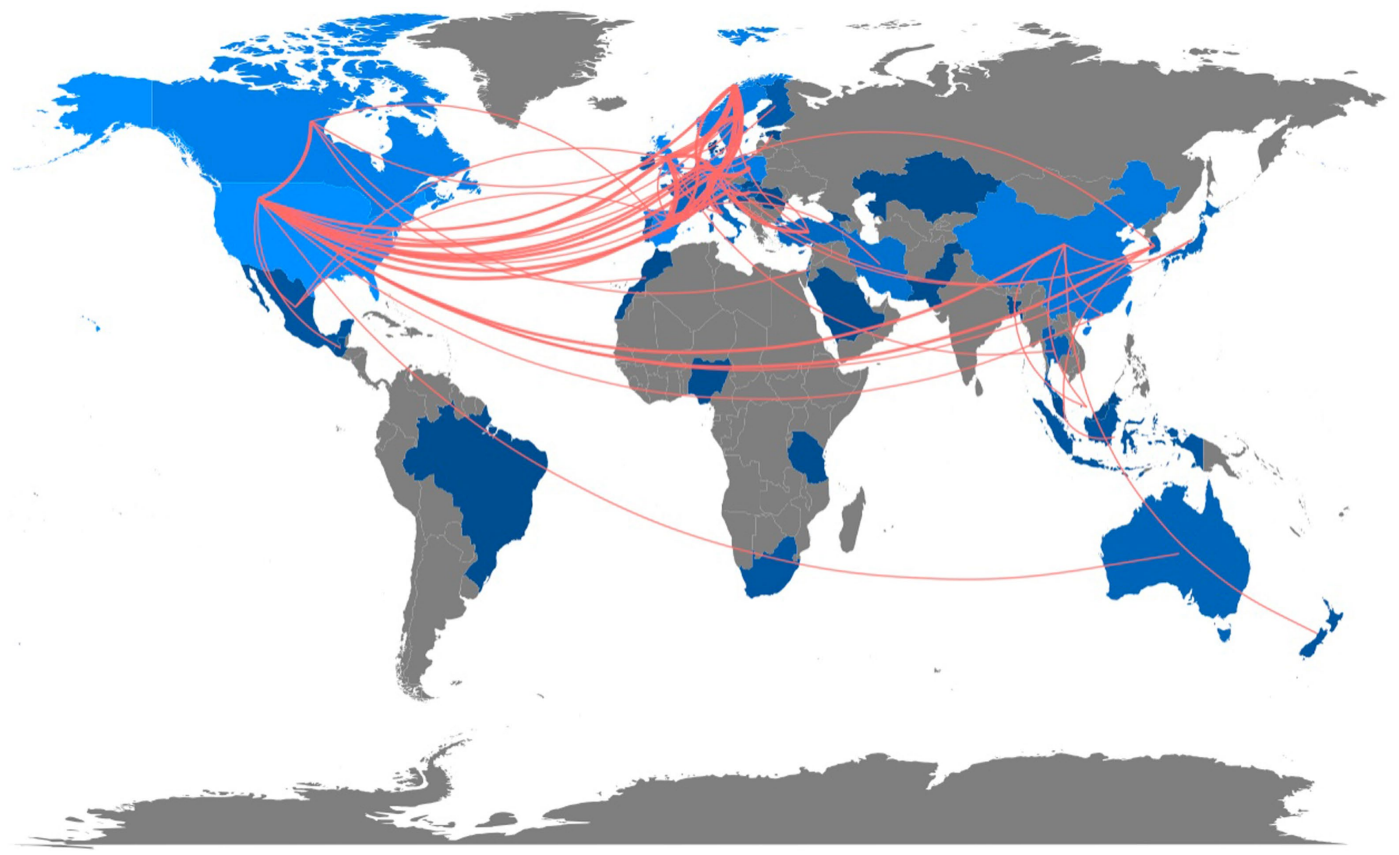
Country collaboration map showing research collaboration among countries (at least once) from 1984 to 2022. Note: Blue indicates contribution, and grey indicates no contribution. Different shades indicate collaboration rates: dark blue without extra lines denotes contribution but no collaboration and light blue represents a high contribution level. Redlines show the path of collaboration.

Regarding international cooperation, Figure 8 illustrates the global collaboration (at least once) map for L3 acquisition worldwide. The map reveals that Germany (*N* = 35) and the United States (*N* = 35) collaborate with other nations the most on L3 acquisition, followed by the United Kingdom (*N* = 34), Norway (*N* = 32), and Spain (*N* = 24). Hence, Europe and the United States are where international scientific collaboration is most prevalent. Several countries, including China and Australia, also collaborate but less frequently with other countries.

**Figure 9:**
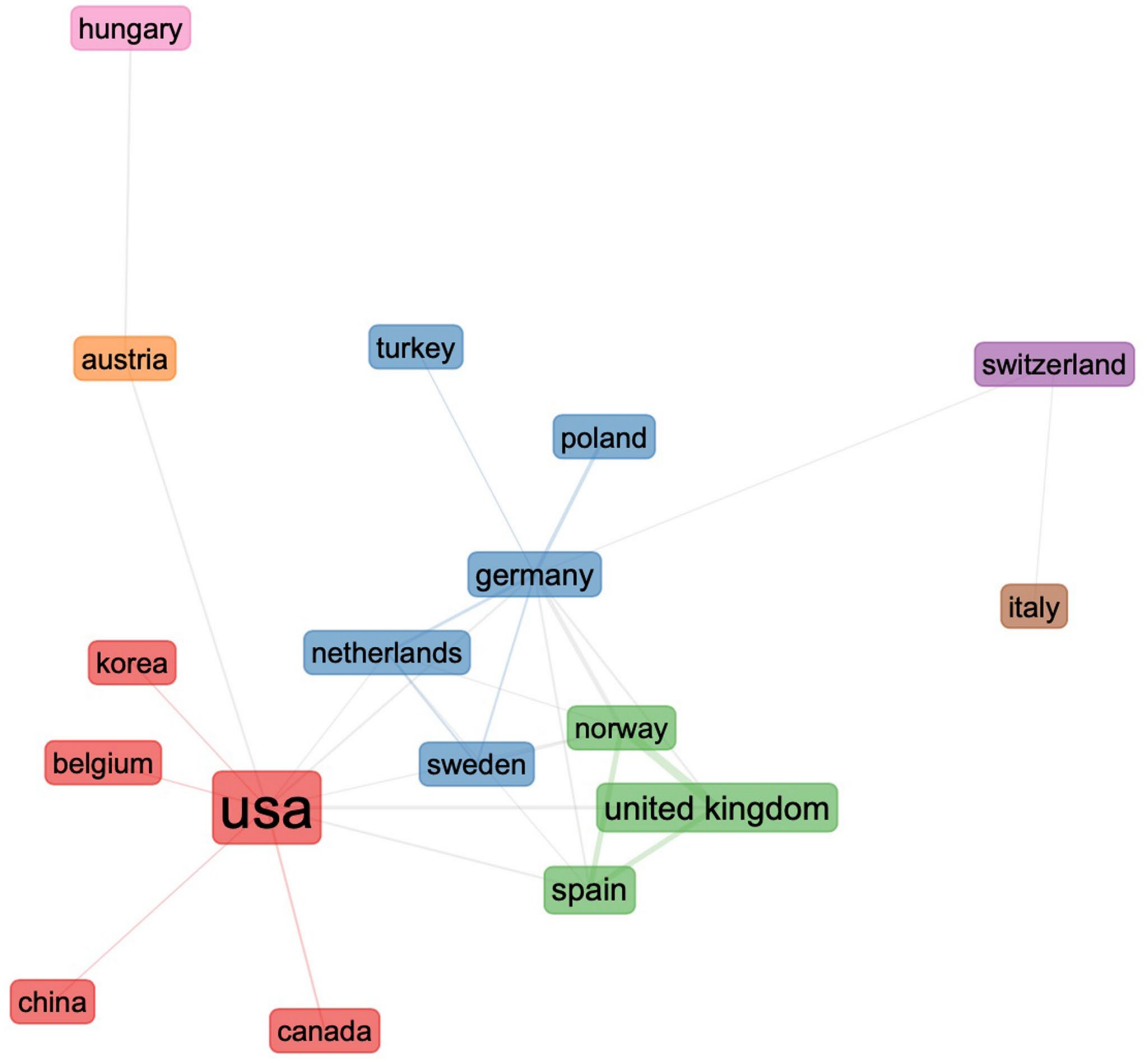
Collaboration between countries.

We set the minimum edge to two to exclude one-time collaboration between countries. Seven groups of cross-national collaboration are depicted in Figure 9. The United States locates at the network’s center and is the most significant node, followed by Germany. Three broad clusters can be distinguished: cluster 1 (in red): the United States, Canada, China, Belgium, and South Korea; cluster 2 (in blue): Germany, Sweden, Netherlands, Poland, and Turkey; and cluster 3 (in green): the United Kingdom, Norway, and Spain.

We sought to examine the collaboration pattern across various nations throughout three time periods. According to the analysis, the collaborative network has expanded significantly during the past four decades. In the initial stage, only the United States and Canada collaborated. In the second, transcontinental (Europe and the United States) and intracontinental collaboration (within Europe) existed. In the third period, the scope of collaboration has grown substantially and reached Asia.

### Trend analysis: Thematic map and evolution

**Figure 10:**
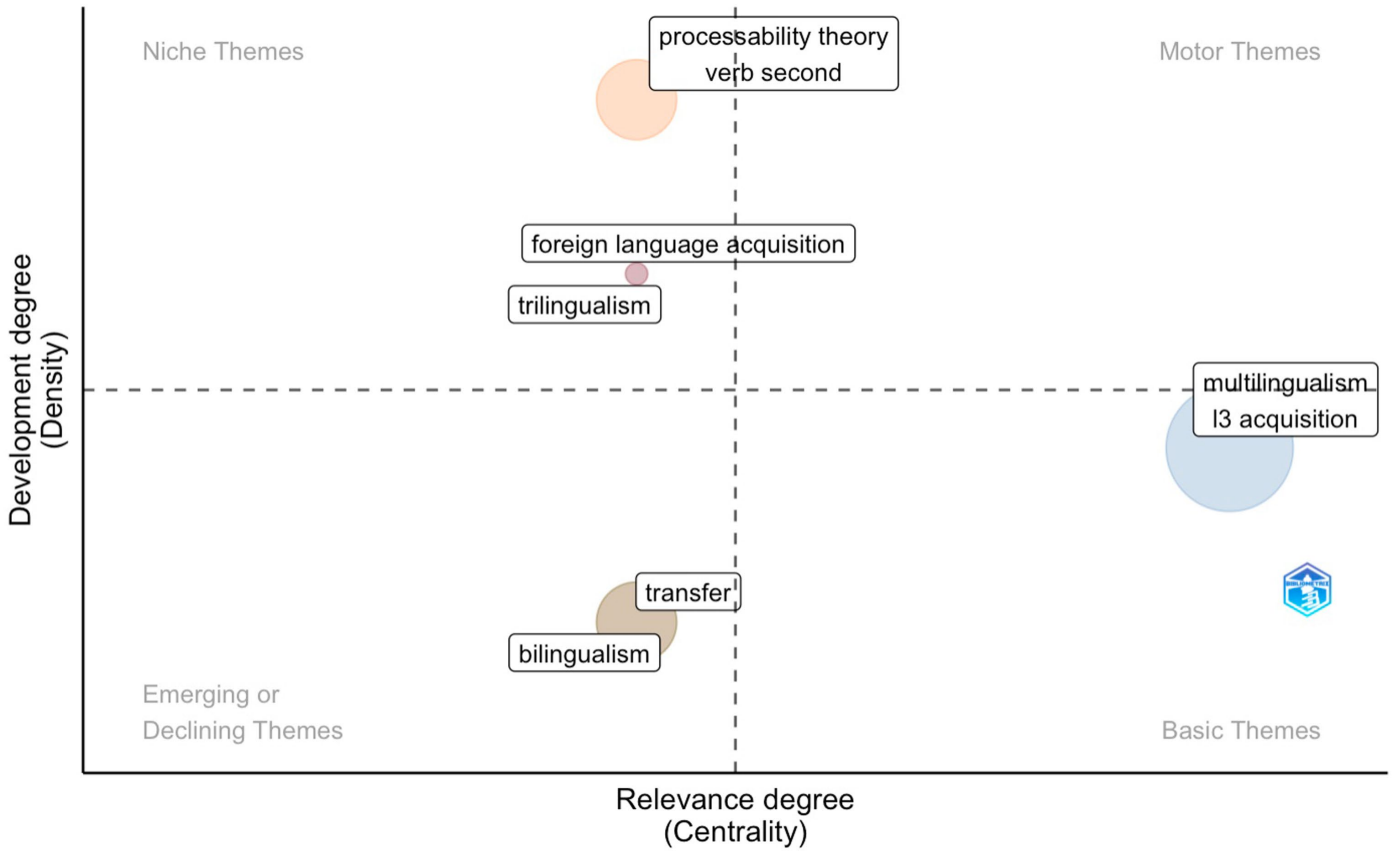
Thematic map showing clusters and the author’s keywords during the first period (1984-2008) identified by the co-occurrence network.

**Figure 11:**
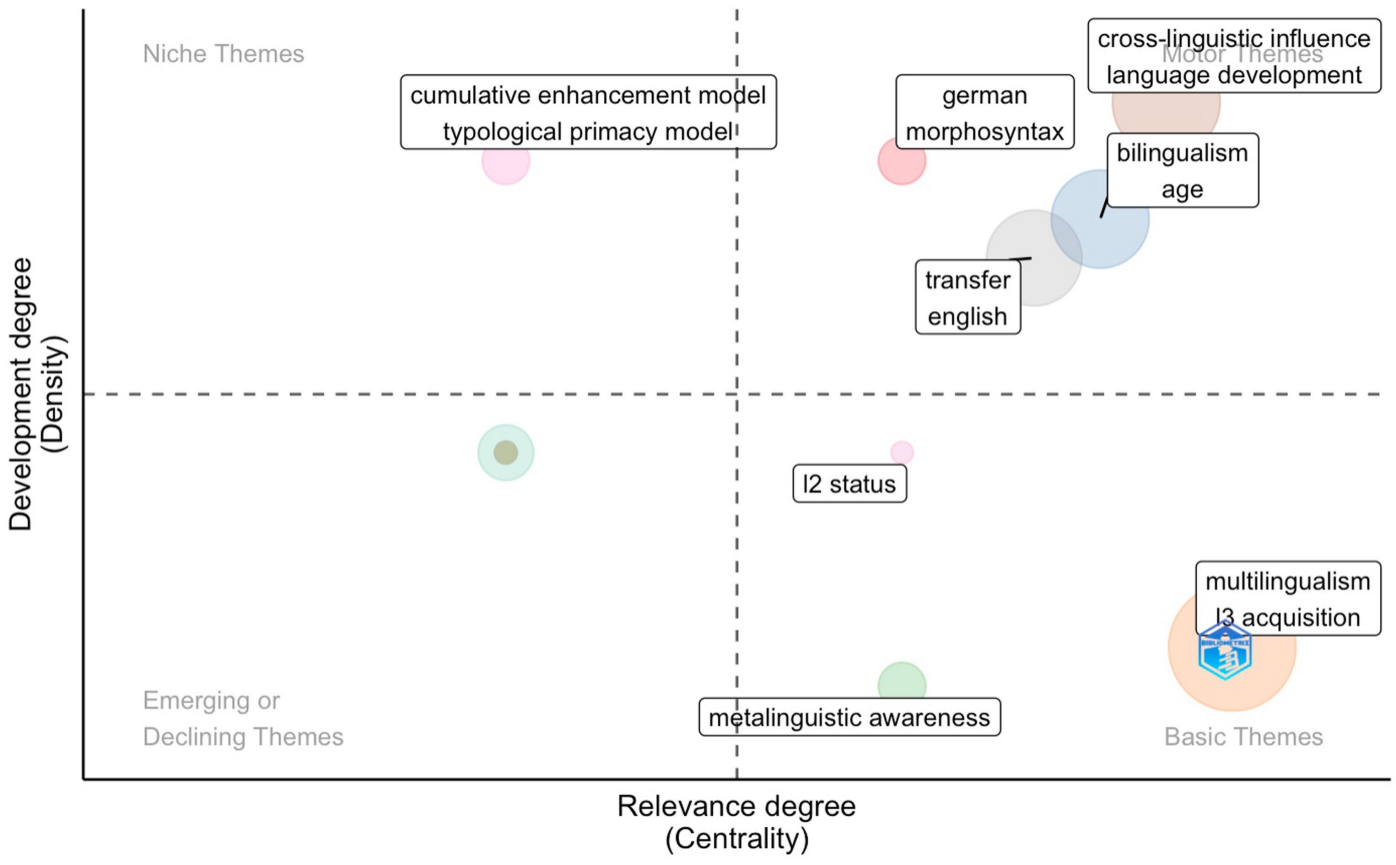
Thematic map showing clusters and the author’s keywords during the second period (2019-2014) identified by the co-occurrence network.

**Figure 12:**
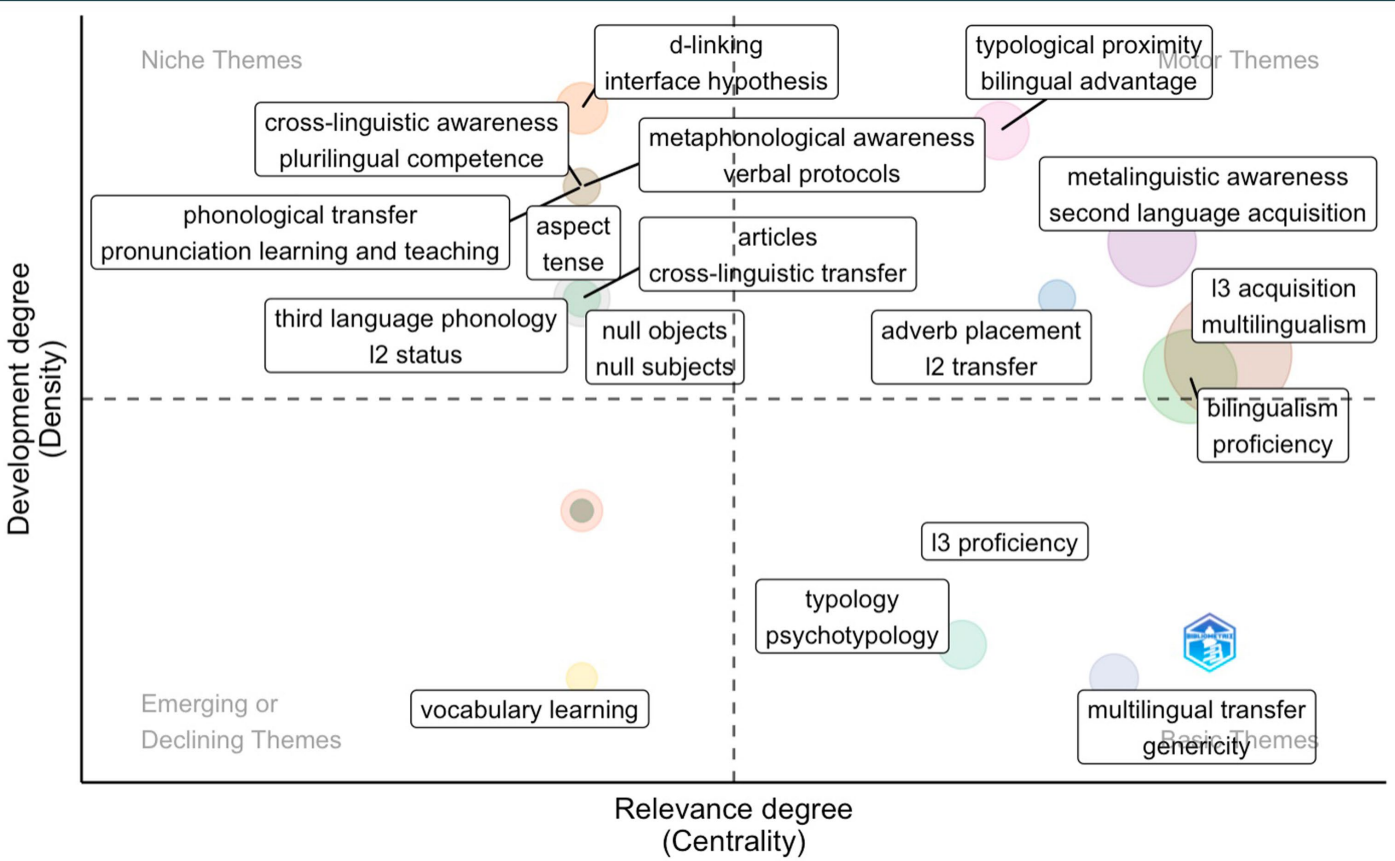
Thematic map showing clusters and the author’s keywords during the third period (2015–2022) identified by the co-occurrence network.

We performed a theme analysis to gain insight into the evolution of the field. To accomplish this, we examine the authors’ keywords over three periods (Figures 10–12) to discover themes, which are categorized by Callon’s density (*Y*-axis) and centrality (*X*-axis) rank values. While conducting the analysis, the following parameters were configured: word minimum frequency was set to 6, the number of words included in the analysis was set to 250, the number of labels for each cluster was set to 2, and a customized stop word list was used to eliminate terms which are irrelevant for this study.

Density denotes the coherence between various nodes, or the intrinsic strength of a theme, whereas centrality quantifies the degree of interconnectedness between different themes (Cobo et al., 2011). In other words, the density implies how well-developed a theme is, and the centrality means the position of this theme within the research field. Hence, the higher the centrality, the more critical the cluster is to the research field (Sorace, 2011). Consequently, the closer a theme is to the right of the *X*-axis or the top of the *Y*-axis, the greater its centrality and density (Cahlik, 2000; Cobo et al., 2011). Moreover, the size of the clusters is determined by the number of occurrences that the keywords contain (Fusco et al., 2020), and different colors are assigned to other clusters by Bibliometrix.

Each figure is further subdivided into four quadrants, which represent different themes. The first quadrant (Q1, motor themes, upper right) is high both in centrality and density, and it features well-developed themes, which are fundamental to the research field and have a high degree of centrality and density. The second quadrant (Q2, niche themes, upper left) is characterized by high density, and it encompasses themes that are also well-developed but specialized and marginal. The third quadrant (Q3, emerging or declining themes, lower left) is low in density and centrality. As suggested by the name, themes located in this area are either just beginning to emerge or are nearing their demise. A rising or decreasing trend in a theme can be identified using a longitudinal examination of a thematic progression (Aria and Cuccurullo, 2017). The fourth quadrant (Q4, basic themes, lower right) covers themes that are vital to the field (high centrality) but still in the early stages of development (low density).

#### Period 1

As Figure 10 shows, in the first period, only a few themes can be observed in general. No motor themes are identified. Two niche themes were located in the second quadrant. One contains *processability theory*, a theory on second language acquisition proposed by Pienemann (1998), and *verb second*, a commonly researched field in syntactic studies. A closer revision of the data suggests that several papers include at least one of the V2 languages (i.e., German, Dutch, Swedish) under study. Other themes contain *foreign language acquisition* and *trilingualism*.

*Bilingualism* and *transfer* emerge in the third quadrant. The term *transfer* stretches back to the 1950s. It is used in Lado (1957) classic study within the contrastive analysis hypothesis, which was first proposed by Fries (1945, p. 19) and further developed by Lado (1957). This approach blends behaviorist theory (psychological aspect) and structuralist theory (linguistic aspect) (Saville-Troike, 2006). According to Odlin (2012), the transfer is ubiquitous in the language. In addition, it may be influenced by linguistic and extralinguistic elements. In the case of linguistic factors, linguistic distance, typology, and syntactic structure must be taken into account; in the case of extralinguistic factors, individual speaker factors, such as proficiency, literacy, linguistic awareness, and social and demographic characteristics, may come into play. The frequent occurrence of *transfer* is also corroborated by the analysis conducted on the most relevant documents, i.e., several L3 studies have centered on the availability of transfer, source of transfer, and quality of the transfer, among others.

Basic themes include *multilingualism* and *L3 acquisition*, indicating that studies on these themes occupy a prominent position in the field but are not yet completely developed.

#### Period 2

Regarding the second period, four clusters were positioned in the first quadrant as motor themes characterized by a high centrality and density, i.e., these themes are essential to the field and are well-developed (Figure 11).

The biggest cluster comprises *cross-linguistic influence* and *language development*. Cross-linguistic influence, a term coined in the mid-80s of the last century (Sharwood Smith, 1983; Kellerman, 1984), is the influence that previously learned languages exert on the acquisition of a new target language (De Angelis, 2007, p. 19). It is one of the central issues in both L2 and L3 acquisition (Cenoz, 2000; Rothman et al., 2019), as the influence in L3 acquisition is not only a bidirectional correlation from one language to another, as it is in second language acquisition, but rather a more complex process (Kellerman and Sharwood-Smith, 1986; De Angelis, 2007, p. 20–21). It is interesting to note that Odlin (2012) comments that the term *transfer*, previously commented, etymologically, is formed of two components, one carry (−fer) and one inter- (cross); in this sense, it transmits the same concept as the term crosslinguistic influence. Kellerman and Sharwood-Smith (1986) defined crosslinguistic influence as the interaction between two languages, one of which was acquired before the new language. In this process of acquisition, influence occurs in both progressive and regressive directions since, on the one hand, the mother tongue (previously acquired language) influences the foreign language (subsequently acquired language) and, on the other hand, the mother tongue modifies its linguistic features due to the influence of the foreign language, resulting in a loss of linguistic knowledge of the L1.

We can also identify a set of themes in the motor themes, already well-studied in the field of L2 acquisition. For instance, *bilingualism* and *age*, *German* and *morphosyntax*, *transfer*, and *English*, can be viewed as a continuation of previous studies on foreign languages in general. In L2 acquisition, the age of acquisition has always been a topic of contention, and this factor is also the subject of research conducted for L3 acquisition. Recall that we have also identified niche topics that point to V2 languages in the first period. Also, in the second period, one of the languages studied was German. Another language that appeared in this stage is English, and given the significance of English in today’s society, the majority of L3 research includes English as one of the languages under study. While we were analyzing the most relevant documents, we found that many studies have focused on the morphosyntactic aspect; this is further corroborated by what we have seen in this period, that morphosyntax appears in the motor themes.

Niche themes start to have some models which account for L3 acquisition, such as the Cumulative Enhancement Model and the Typological Primacy Model. The second period extends from 2009 to 2014, and both models were proposed within this time frame.

*Multilingualism* and *L3 acquisition* remain within the basic themes, i.e., they are still developing. However, it is noticeable that *metalinguistic awareness* and *L2 status* emerge in the basic themes. Metalinguistic awareness is among the advantages that, according to Cenoz (2013, p. 75), bilingual speakers have the edge over monolingual speakers in acquiring a foreign language. She condenses these benefits into three criteria. (1) metalinguistic awareness, (2) more learning mechanisms, and (3) a more extensive linguistic repertoire. Monolingual speakers differ from bilingual speakers in the learning process (Thomas, 1988; Cenoz and Genesee, 1998; De Angelis, 2005; Safont-Jordà, 2011), as the latter already know another language, i.e., more than one linguistic inventory (Nayak et al., 1990; Bialystok et al., 2004; Tremblay and Sabourin, 2012; Cenoz, 2013).

#### Period 3

With the bourgeoning of research in L3 acquisition, the third period contains more themes than the previous two periods (Figure 12).

In the first quadrant (motor themes), the biggest cluster is formed by *L3 acquisition* and *multilingualism*. These two themes have moved from basic themes (period 1 and period 2) to motor themes (period 3), suggesting that they are both well-developed and situated at a central position in the research field. In this quadrant, we can identify more themes related directly to L3 acquisition, such as *typological proximity*, *bilingual advantage*, and *L2 transfer*.

In the niche themes, key concepts in syntax are also found (*aspect*, *tense*, *d-linking*, *interface hypothesis*, *null objects*, and *null subjects*). Notably, although themes in the first two periods focused more on morphosyntax, phonology began to gain prominence in the third period. Themes related to phonology and pronunciation also appear in this quadrant (*phonological transfer*, *pronunciation learning*, *teaching*, *third language phonology*). Moreover, Niche themes also start to encompass themes related to multilingual learners’ advantages, such as *cross-linguistic awareness*, *plurilingual competence*, and *metaphonological awareness*. It needs to be pointed out that *L2 status* has moved from basic theme to niche theme, suggesting that it is better developed but less central.

In the emerging or declining themes, we found *vocabulary learning*. A revision of the data suggests that this theme is fading. This runs in line with Lin and Lei (2020) observation that vocabulary knowledge is on the decline. The authors explained that more attention was drawn to deeper cognition levels, such as metalinguistic awareness.

In basic themes, we can find *L3 proficiency*, *(psycho)typology*, *multilingual transfer*, and *genericity*, suggesting that studies also examine factors that contribute to the crosslinguistic influence, such as language typology, psychotypology, and proficiency.

## Discussion

In the present study, we set out to examine the literature on L3 acquisition for nearly four decades (1984–2022) through changes in influential sources, authors, institutions, and countries, as well as the collaboration pattern and hot research spots. The bibliometric analysis in this study provides a comprehensive overview of the state-of-art L3 acquisition.

With regard to the first research question, in the past 40 years, thanks to an increasing number of contributing researchers, sources, institutions, and countries, L3 acquisition has witnessed a rapid and thriving expansion: 1984–2008 as the infancy, 2009–2014 as the steady development, and 2015–2022 as the burst development stage. Besides the natural growth of this discipline, the expanding body of research on L3 acquisition may also be attributable to the international conferences and workshops that have fostered academic collaboration and exchange. Third Language Acquisition, Satellite Workshop on “Phonetics and Phonology in Third Language Acquisition” at the 16th International Congress of Phonetic Sciences (2007), to name a few. Moreover, numerous books focusing on L3 are released, and journals such as the *International Journal of Multilingualism* have published several L3-focused special issues.

As for the second research question, regarding the publishing sources, our study on L3 acquisition seems to have identified different scenarios in terms of the most significant sources from Lin and Lei (2020) and Zhang (2020). A plausible explanation is that L3 research shares similarities with second language acquisition and bi−/multilingualism but also presents some uniqueness. As put by Cenoz and Hoffmann (2003, p. 2), the rising body of research on third language acquisition and multilingualism is a response to the need to identify the distinctions between third language acquisition and second language acquisition, and between multilingualism and bilingualism. For instance, L3 learners have access to more language experience than L2 learners (Cenoz, 2003). Hence, focusing solely on L2 speakers might be too restrictive, given the linguistic landscape in today’s society (De Angelis, 2007). Similarly, Hoffmann notes that while trilingualism shares commonalities with both bilingualism and multilingualism, it also possesses several unique properties (Hoffmann, 2001). Therefore, L3 acquisition is not just an extension of other previously well-established fields, such as second language acquisition or bilingualism (De Angelis, 2007, p. 3), but a unique research space waiting for more attention and exploration.

Of all sources, the *International Journal of Multilingualism* has remained steady throughout the three periods, occupying a dominant position among all sources. The significance of the *International Journal of Multilingualism* to L3 acquisition can be understood given the aim of the journal, which states: “The aim of the International Journal of Multilingualism (IJM) is to foster, present, and spread research focused on psycholinguistic, sociolinguistic and educational aspects of multilingual acquisition and multilingualism. The journal is interdisciplinary and seeks to go beyond bilingualism and second language acquisition by understanding the specific characteristics of acquiring, processing, and using more than two languages.” (Aims of the *International Journal of Multilingualism* 2022). Moreover, as affirmed by de Bot (de Bot, 2015, p. 78), with its own journal, *International Journal of Multilingualism*, books, and conferences devoted to this topic, the L3 community has expanded significantly during the past decade.

In terms of authors, the field of L3 acquisition has witnessed an increase in the number of researchers devoted to this research area. Recall that there were only 63 researchers in this field in the first period, which increased to 171 in the second and 428 in the third. In general, names that appeared across the three periods vary greatly. However, we also found that some scholars have worked in this field for over three decades, whereas the field of L3 acquisition also abreast new scholars.

Of the most prolific institutions and countries, the University of the Basque Country is the most productive affiliation, followed by The Arctic University of Norway, Stockholm University, and Adam Mickiewicz University. All countries appeared among the top 10 productive affiliations belonging to European or Anglo-Saxon countries. Similar results are obtained in studies (Fusco et al., 2020) with a dominant role of Anglo-Saxon and European countries in publication.

The data in our study has shown that Spain seems to be the leading country in terms of the number of studies published, and the majority of countries that appeared on the top list are European nations. The United States’ advantage over other countries in terms of number of publications does not appear to be as salient as other studies have deemed, for instance, Zhang (Zhang, 2020) found that more than 55% of the documents published in the top SLA journals were affiliated with universities in the US. Other countries, such as China, are becoming increasingly significant in the sense that more papers are published by authors from these countries (32 papers). Other studies also suggest that the US tends to occupy the dominant position in the academic circle but shows a slow decline, with the publications from other emerging powerhouses (e.g., China, United Kingdom, Australia, Malaysia, South Africa, Indonesia) leading a steady increase (Liao and Lei, 2017; Lei and Liu, 2019a; Huan and Guan, 2020; Wang et al., 2022), providing some evidence to the globalization (Hyland and Jiang, 2021). Similarly, Pearson (Pearson, 2022) reported a well-represented North American scholarship across academic output in Teaching English as a Second Language Electronic Journal, particularly in studies contextualized in higher education. The reason European countries such as Spain have a more substantial presence in L3 acquisition (most productive affiliations) might be due to the linguistic environment and policies in these regions. For instance, the majority of research on L3 acquisition from Spain is conducted in the Basque Country and Catalonia, two regions with similar socio-educational backgrounds where Spanish and the minority language (Basque or Catalan) are taught in schools (Cenoz, 2003). Traditional English-speaking countries, such as the US, might have traditionally lagged in promoting learning a foreign language, particularly in learning multiple foreign languages (Rothman et al., 2019).

The analysis of the most relevant documents in terms of citation points out several interesting observations. Numerous studies are approached from psycholinguistic (bi−/multilingualism), linguistic (i.e., syntax), sociolinguistic (language use), and educational perspectives, similar to what has been proposed by (Cenoz and Hoffmann, 2003, p. 2). The study on L3 acquisition shares both similarities and differences between second language acquisition and multilingualism. Nevertheless, multiple studies (Cenoz, 2000, 2001, 2003; Gallardo del Puerto, 2007) suggest that multilingual acquisition is more complex than second language acquisition, despite the similarities. Cenoz (2000, p. 47) explains that the difficulty of multilingual learning is due, on the one hand, to the speaker’s unique characteristics and, on the other hand, to the process of second and third language acquisition. According to Falk and Bardel (2010), potential interactions between the language systems in the mind of the multilingual speaker and their access to universal grammar are critical differences in this learning process.

The most relevant documents also point out some models that account for transfer in acquiring a third language. The L2 status factor acknowledges a more significant impact from the L2 in acquiring an L3 (Falk and Bardel, 2010, 2011). As suggested by its name, the Cumulative Enhancement model considers that the effect of the previously learned languages on an L3 is cumulative and, hence, are all possible origins of transfer (Flynn et al., 2004). The Typological Primacy Model also acknowledges the possible transfer from both L1 and L2 and proposes a decisive role of perceived proximity in the transfer (Rothman, 2011, 2015). Similarly, the Scalpel Model (Slabakova, 2017) views the possible transfer from both L1 and L2 but considers that the facilitative parameter or value will be transferred with scalpel-like precision. The Linguistic Proximity Model, similar to the previous ones, also finds a possible transfer from all languages a learner dominates to the one currently acquiring (Westergaard et al., 2017). Still, the source of transfer is conditioned by the structural similarities between all languages. All of these models represent an attempt by researchers to respond to the various phenomena seen in L3 studies, and they have significantly enriched the field of L3 acquisition.

The third research question addresses the collaborative pattern among different research constituents. Collaboration between authors, countries, and institutions rises as time passes, which is vital in promoting international communication. Some previous studies found a low collaborative rate in language journals (Dong et al., 2022). Although the collaboration in the linguistic field might be less than in the scientific field, in our study, we must acknowledge a rising trend: as time passes, an increasing number of scholars, institutions, and countries develop a network by interacting intensively in academic journals. This “invisible college” (Crane, 1969), which mimics a social circle, might integrate less productive researchers into a more extensive network of influence and increase engagement and communication. The collaboration between different research consitituents may also be motivated by academic and pratical considerations, such as the migration and the mobility of scholars and Ph.D. candidates, which may facilitate the formation of research collaborations between home universities and other researchers overseas. In addition, we found that the primary collaboration occurs among European and Anglo-Saxon countries, whereas cooperation between developing countries seems to be less. However, there is a general trend of extension from more European-based collaboration to a global collaboration, which may benefit the scientific world, for instance, through more visibility and high citation (Mohsen, 2021).

The fourth question is related to the thematic trend across different periods. The results obtained from the thematic analysis provide some benefits: we identified some research hotspots (i.e., *transfer*, *crosslinguistic influence*) and viewed some changes in research topics and foci in L3 acquisition, which is to be expected in a nascent and rapidly increasing research field. More topics are streaming into the field, and scholars’ interests are expanding to encompass multiple perspectives. Our results seem similar to what De Angelis (2007) has claimed, that research on the cognitive and psycholinguistic aspects of multilingualism appears slower than that on the sociolinguistic and educational aspects. Much research in this study has focused on the transfer, with substantial attention devoted to morphosyntax, less on other parts of the linguistic field. This observation was also made by Cabrelli Amaro (2012) that the area of L3 acquisition has witnessed a sharp increase in the domain of L3/Ln acquisition of morphosyntax; the linguistic study of L3 acquisition in other aspects is still understudied, for example, in L3 phonology. However, in the third period, the emergence of phonology-related themes was observed. Overall, the changes in research foci seem to suggest a move towards a broader scope of L3 acquisition. In our study, the thematic maps prove helpful in tracking the development of a research field to grasp its evolution. Nevertheless, it should be emphasized that the thematic map analysis is performed based on the author’s keywords, which does not preclude the presence of other lines of research that, due to insufficient development, did not cluster into a different topic.

## Conclusion

The present study employed the bibliometric method to observe the evolutionary path, research constituents, collaborative patterns, and research trends in the field of L3 acquisition. The results suggest that the evolution of L3 acquisition may be divided into three phases, with 1984 to 2008 as the initiation phase, 2009 to 2014 as the development phase, and 2015 to present as the burst phase. In terms of the most productive publication venue, *The International Journal of Multilingualism* seems to be the most stable source which contributes to this field. Some experienced and novate scholars are identified across different periods, as well as the most prolific institutions and countries. Concerning the collaborative pattern, more collaboration is found over time, and the collaboration has extended from European and Anglophone countries to other continents all over the world. The research hotspots have enriched over time and continue to expand.

Despite efforts to conduct the bibliometric analysis in the most effective and precise manner possible, this study has several limitations. This study analyzed only Scopus-indexed documents published between 1984 and 2022. Consequently, there is a possibility that it is not exhaustive since there may be relevant articles on L3 acquisition that are not included in the Scopus database. Despite this, our research indicates that Scopus has a more extensive coverage than WoS on this topic. In addition, documents retrieved on Scopus only start from 1984; therefore, this study does not provide a thorough summary of real data. This, however, is a general restriction, as no scientific database is complete, and each has its strengths and weaknesses (Falagas et al., 2008). Also, given the limitation in analysis tools (some analysis only is available for English), in this study, unfortunateley no publications in other languages, e.g., German, French, and Spanish, are included in this study. In their review of papers published in German, Marx and Hufeisen (2004) noted that research published in other languages but not English is frequently ignored by academics. However, this does not mean that these studies are irrelevant to L3 learning. Future research can investigate studies related to L3 published in other languages. The second limitation relates to the indices that have been the subject of our bibliometric analysis. In this study, we have only focused on a limited set of indices. Still, there is a vast array of bibliometric parameters available for selection. There are aspects we cannot cover due to space constraints, i.e., co-citation analysis and bibliometric coupling. Despite this, and even though the number of items included in this study may not fully reflect the whole picture of L3 acquisition, the results provide insights into the evolving trends in L3 acquisition studies’ future research direction, as well as data to assist additional scholars and editors in selecting topics of emerging and substantial interest in L3 acquisition.

## Data availability statement

The raw data supporting the conclusions of this article will be made available by the authors, without undue reservation.

## Author contributions

The author confirms being the sole contributor of this work and has approved it for publication.

## Funding

The research was funded by Fundamental Research Funds for the Central Universities (East China Normal University), grant numbers [No. 2021 ECNU-HWCBFBLW001], [No. 43800-20101-222201], [No. 43800-20101-222352] and [No. 43800-20101-222087].

## Conflict of interest

The author declares that the research was conducted in the absence of any commercial or financial relationships that could be construed as a potential conflict of interest.

## Publisher’s note

All claims expressed in this article are solely those of the authors and do not necessarily represent those of their affiliated organizations, or those of the publisher, the editors and the reviewers. Any product that may be evaluated in this article, or claim that may be made by its manufacturer, is not guaranteed or endorsed by the publisher.
