# Supplementary material for: Mapping the research trends of third language acquisition: A bibliometric analysis based on Scopus
Source: Front Psychol. 2022 Nov 3;13:1021517. doi: 10.3389/fpsyg.2022.1021517 (PMC9670133; doi:10.3389/fpsyg.2022.1021517)
Supplement: Supplementary file 1 [file Table_1.DOCX]

Supplementary Material

## Supplementary Tables and Figures

**Supplementary Table 1.** Main information about the collection.

|  | **Description** | **Results** |
| --- | --- | --- |
| **Main information** | Timespan | 1984: 2022 ^2^ |
|  | Sources | 157 |
|  | Documents | 425 |
|  | Document Average Age | 6.68 |
|  | Average citations per doc | 16.36 |
|  | Author’s Keywords | 966 |
| **Authors** | Authors | 605 |
|  | Author per Document | 1.43 |
|  | Authors of single-authored docs | 135 |
| **Authors collaboration** | Single-authored docs | 188 |
|  | Co-Authors per Doc | 2.04 |
|  | International co-authorships % | 17.88 |

**Supplementary Table 2.** Number of studies per data type and language during data retrievement.

|  | **Type** | ***N*** |
| --- | --- | --- |
| **Data Type** | article | 348 |
|  | book | 10 |
|  | book chapter | 33 |
|  | conference paper | 25 |
|  | editorial | 2 |
|  | erratum | 1 |
|  | review | 19 |
|  | note | 6 |
|  | short survey | 2 |
|  | Total | 446 |
| **Data Language** | Afrikaans | 1 |
|  | English | 417 |
|  | English; Croatian | 2 |
|  | English; Dutch | 1 |
|  | English; French | 3 |
|  | English; German | 1 |
|  | English; Spanish | 1 |
|  | Estonian | 1 |
|  | French | 3 |
|  | German | 4 |
|  | Malay | 1 |
|  | Portuguese | 1 |
|  | Russian | 1 |
|  | Spanish | 5 |
|  | Total | 442 |

**Supplementary Table 3.** Top 5 publications across the three periods and the number of publications.

|  | Period 1 | | Period 2 | | Period 3 | |
| --- | --- | --- | --- | --- | --- | --- |
| Ranking | Source | N | Source | N | Source | N |
| 1 | International Journal of Multilingualism | 11 | International Journal of Multilingualism | 25 | International Journal of Multilingualism | 27 |
| 2 | Journal of Multilingual and Multicultural Development | 3 | Second Language Learning and Teaching | 11 | Second Language Research | 18 |
| 3 | Second Language Research | 3 | Second Language Research | 8 | International Journal of Bilingualism | 15 |
| 4 | Inquiries In Linguistic Development:  In Honor of Lydia White | 2 | International Review of Applied Linguistics in Language Teaching | 7 | Bilingualism | 12 |
| 5 | International Journal of Bilingualism | 2 | Third Language Acquisition  and Universal Grammar | 6 | Linguistic Approaches to Bilingualism | 11 |
|  | International Journal of the Sociology of Language | 2 |  | | | |
|  | Language Awareness | 2 |  |  |  |  |
|  | Language Learning | 2 |  |  |  |  |

**Supplementary Table 4.** Top-10 productive sources, number of documents. Journal’s appearances and ranking as the 15 most influential journals in Zhang (Zhang, 2020) and the 20 most influential journals in Lin and Lei (Lin and Lei, 2020) are also provided.

| Sources | N | Scopus Categories | Zhang (2020) | Lin and Lei (2020) |
| --- | --- | --- | --- | --- |
| International Journal of Multilingualism | 63 | Linguistics and Language |  |  |
| Second Language Research | 29 | Linguistics and Language & Education | Yes |  |
| International Journal of Bilingualism | 21 | Linguistics and Language & Education |  | 3 |
| International Review of Applied Linguistics in Language Teaching | 14 | Linguistics and Language |  |  |
| Linguistic Approaches to Bilingualism | 14 | Linguistics and Language |  | 9 |
| Bilingualism: Language & Cognition | 13 | Linguistics and Language & Education | Yes | 1 |
| Second Language Learning and Teaching | 13 | Linguistics and Language & Education |  |  |
| International Journal of Bilingual Education and Bilingualism | 12 | Linguistics and Language & Education |  | 2 |
| Journal of Multilingual and Multicultural Development | 10 | Linguistics and Language & Education &Cultural Studies |  | 4 |
| Language Awareness | 10 | Linguistics and Language & Education |  |  |


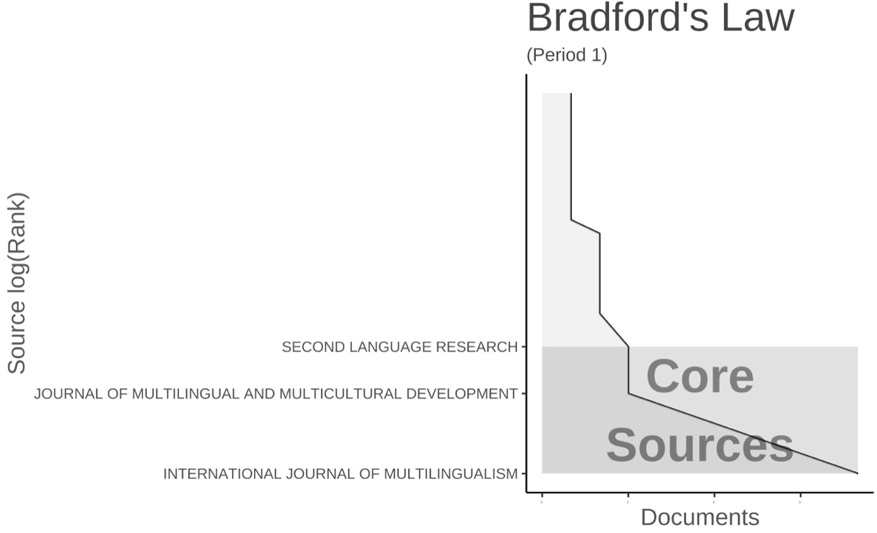


**Supplementary Figure 1.** Source clustering through to Bradford’s Law in period 1.


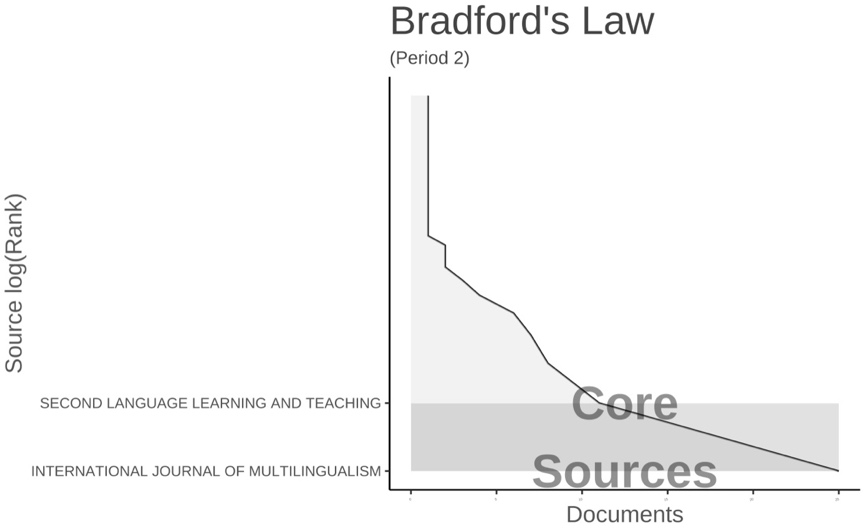


**Supplementary Figure 2.** Source clustering through to Bradford’s Law in period 2.


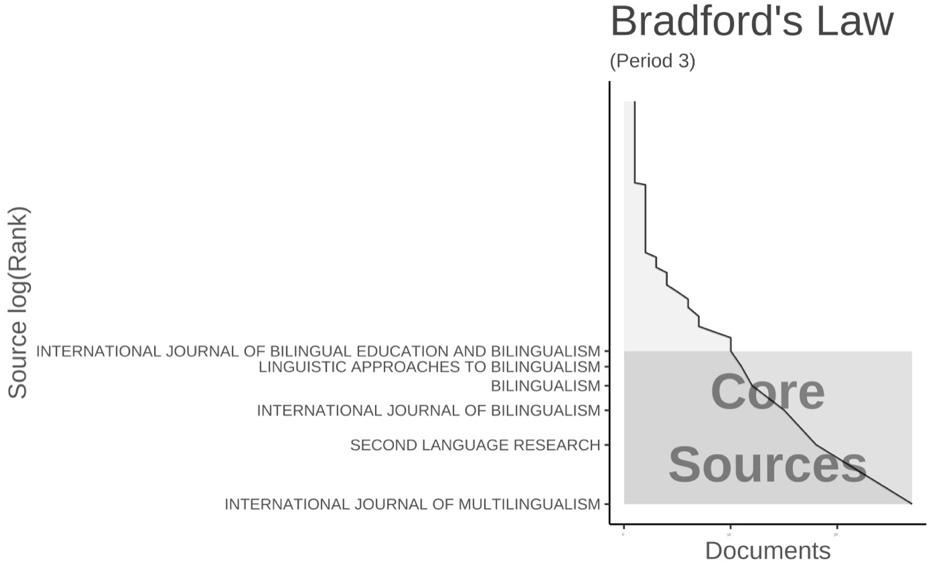


**Supplementary Figure 3.** Source clustering through to Bradford’s Law in period 3.

**Supplementary Table 5.** Top 5 sources across the three periods, impact indexes, number of total citations and number of publications (NP).

| **Period** | **Source** | **h_index** | **Total Citation** | **NP** |
| --- | --- | --- | --- | --- |
| Period1 | International Journal of Multilingualism | 9 | 432 | 11 |
|  | Journal of Multilingual and Multicultural Development | 3 | 112 | 3 |
|  | Second Language Research | 3 | 222 | 3 |
|  | Inquiries inLinguistic Development: inHonor of Lydia White | 2 | 26 | 2 |
|  | International Journal of Bilingualism | 2 | 282 | 2 |
|  | International Journal of The Sociology of Language | 2 | 63 | 2 |
|  | Language Awareness | 2 | 153 | 2 |
|  | Language Learning | 2 | 330 | 2 |
| Period2 | International Journal of Multilingualism | 15 | 572 | 25 |
|  | Second Language Research | 8 | 550 | 8 |
|  | IRAL - International Review of Applied Linguistics in Language Teaching | 6 | 286 | 7 |
|  | Second Language Learning and Teaching | 5 | 52 | 10 |
|  | Third Language Acquisition and Universal Grammar | 5 | 101 | 6 |
| Period3 | International Journal of Bilingualism | 8 | 303 | 14 |
|  | International Journal of Multilingualism | 8 | 150 | 17 |
|  | Bilingualism | 7 | 304 | 12 |
|  | Language Awareness | 5 | 67 | 7 |
|  | Second Language Research | 5 | 126 | 18 |

**Supplementary Table 6.** Author Productivity through Lotka’s Law by period.

| **Period** | **Documents written** | **N. of Authors** | **Proportion of Authors** |
| --- | --- | --- | --- |
| All | 1 | 485 | 0.802 |
|  | 2 | 69 | 0.114 |
|  | 3 | 24 | 0.04 |
|  | 4 | 10 | 0.017 |
|  | 5 | 3 | 0.005 |
|  | 6 | 4 | 0.007 |
|  | 7 | 4 | 0.007 |
|  | 8 | 1 | 0.002 |
|  | 9 | 3 | 0.005 |
|  | 10 | 1 | 0.002 |
|  | 21 | 1 | 0.002 |
| 1 | 1 | 55 | 0.873 |
|  | 2 | 4 | 0.063 |
|  | 3 | 3 | 0.048 |
|  | 4 | 1 | 0.016 |
| 2 | 1 | 145 | 0.853 |
|  | 2 | 20 | 0.118 |
|  | 3 | 3 | 0.018 |
|  | 4 | 1 | 0.006 |
|  | 7 | 1 | 0.006 |
| 3 | 1 | 348 | 0.813 |
|  | 2 | 50 | 0.117 |
|  | 3 | 14 | 0.033 |
|  | 4 | 7 | 0.016 |
|  | 5 | 1 | 0.002 |
|  | 6 | 2 | 0.005 |
|  | 7 | 3 | 0.007 |
|  | 8 | 1 | 0.002 |
|  | 9 | 1 | 0.002 |
|  | 14 | 1 | 0.002 |


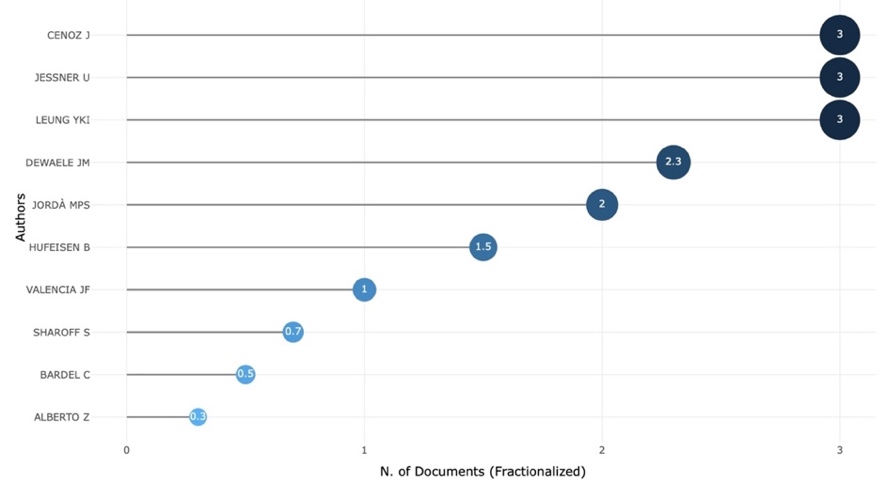


**Supplementary Figure 4.** Most relevant authors according to the fractionalized number of documents (period 1).


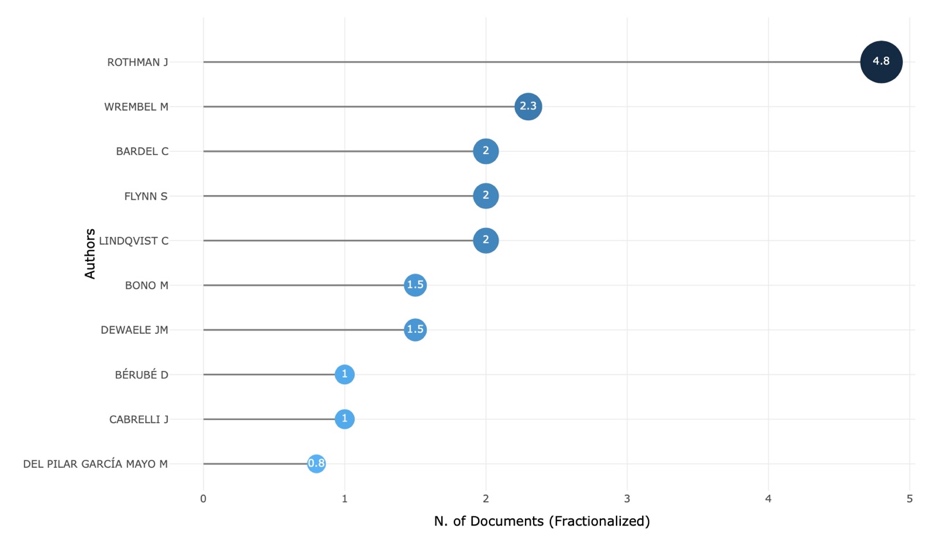


**Supplementary Figure 5.** Most relevant authors according to the fractionalized number of documents (period 2).


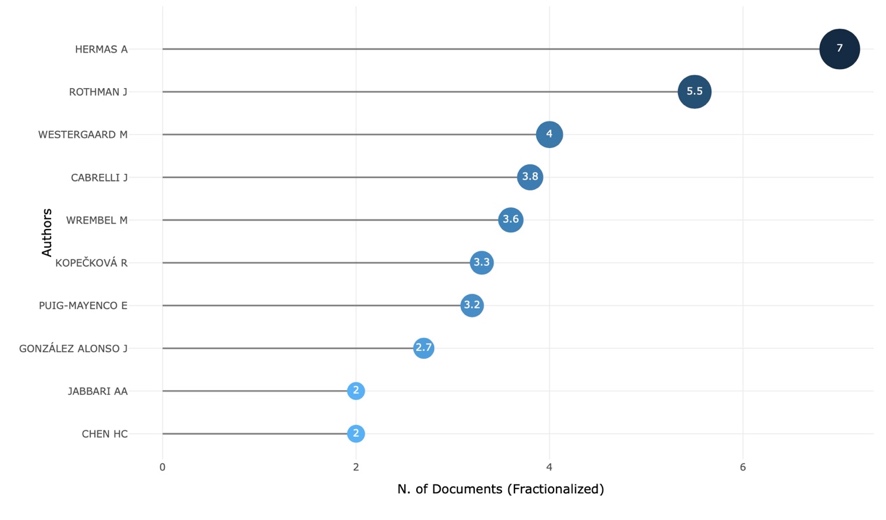


**Supplementary Figure 6.** Most relevant authors according to the fractionalized number of documents (period 3).


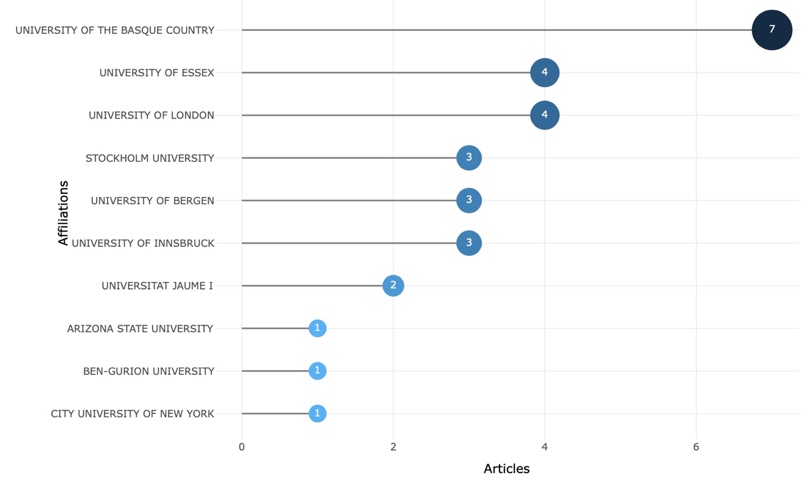


**Supplementary Figure 7.** Most relevant affiliations according to the fractionalized number of documents (period 1).


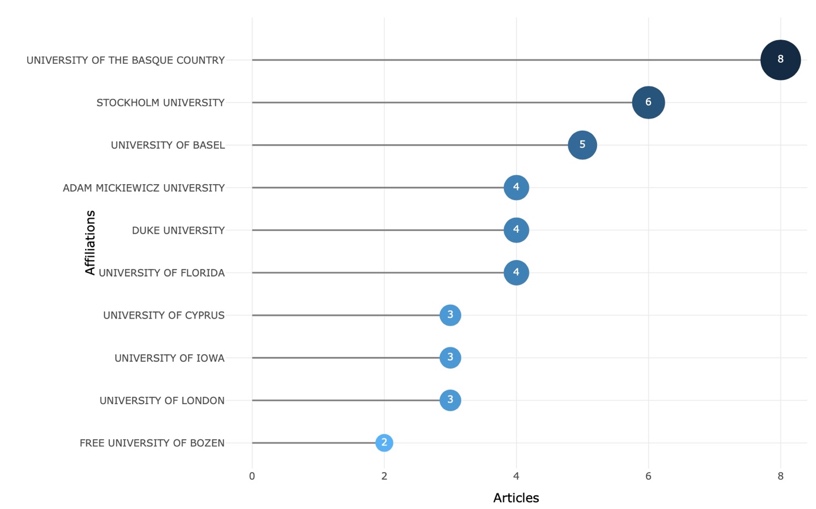


**Supplementary Figure 8.** Most relevant affiliations according to the fractionalized number of documents (period 2).


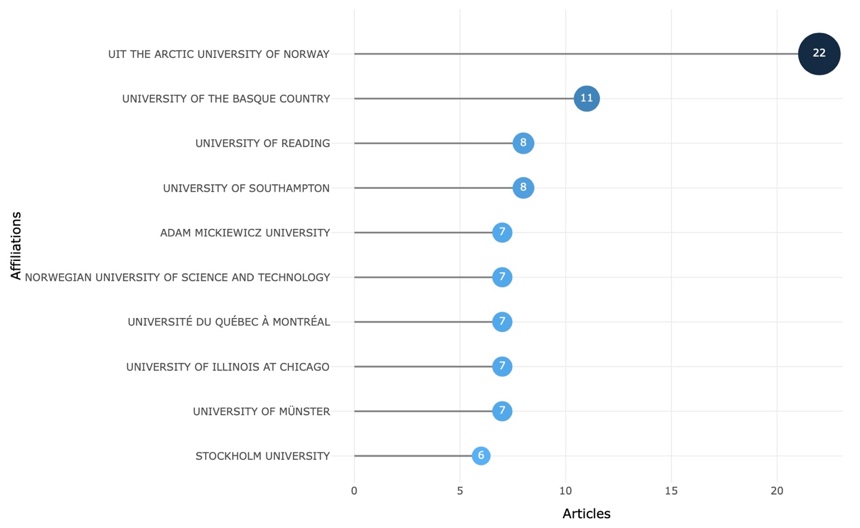


**Supplementary Figure 9.** Most relevant affiliations according to the fractionalized number of documents (period 3).

**Supplementary Table 7.** Country’s Scientific Production.

| Region | Production |
| --- | --- |
| USA | 125 |
| Spain | 60 |
| Germany | 56 |
| UK | 53 |
| Canada | 42 |
| Norway | 40 |
| China | 32 |
| Poland | 31 |
| Sweden | 21 |
| Switzerland | 21 |
| Austria | 14 |
| Netherlands | 14 |
| Iran | 13 |
| France | 9 |
| Israel | 9 |
| Japan | 9 |
| Italy | 8 |
| Portugal | 8 |
| Turkey | 8 |
| South Korea | 7 |
| Australia | 6 |
| Belgium | 6 |
| Singapore | 6 |
| Thailand | 6 |
| Cyprus | 5 |
| Croatia | 3 |
| Finland | 3 |
| Hungary | 3 |
| Ireland | 3 |
| Kazakhstan | 3 |
| Mexico | 3 |
| New Zealand | 3 |
| Indonesia | 2 |
| Saudi Arabia | 2 |
| South Africa | 2 |
| Bangladesh | 1 |
| Brazil | 1 |
| Denmark | 1 |
| Estonia | 1 |
| Georgia | 1 |
| Guatemala | 1 |
| Luxembourg | 1 |
| Malaysia | 1 |
| Morocco | 1 |
| Nigeria | 1 |
| Pakistan | 1 |
| Romania | 1 |
| Slovakia | 1 |
| Tanzania | 1 |


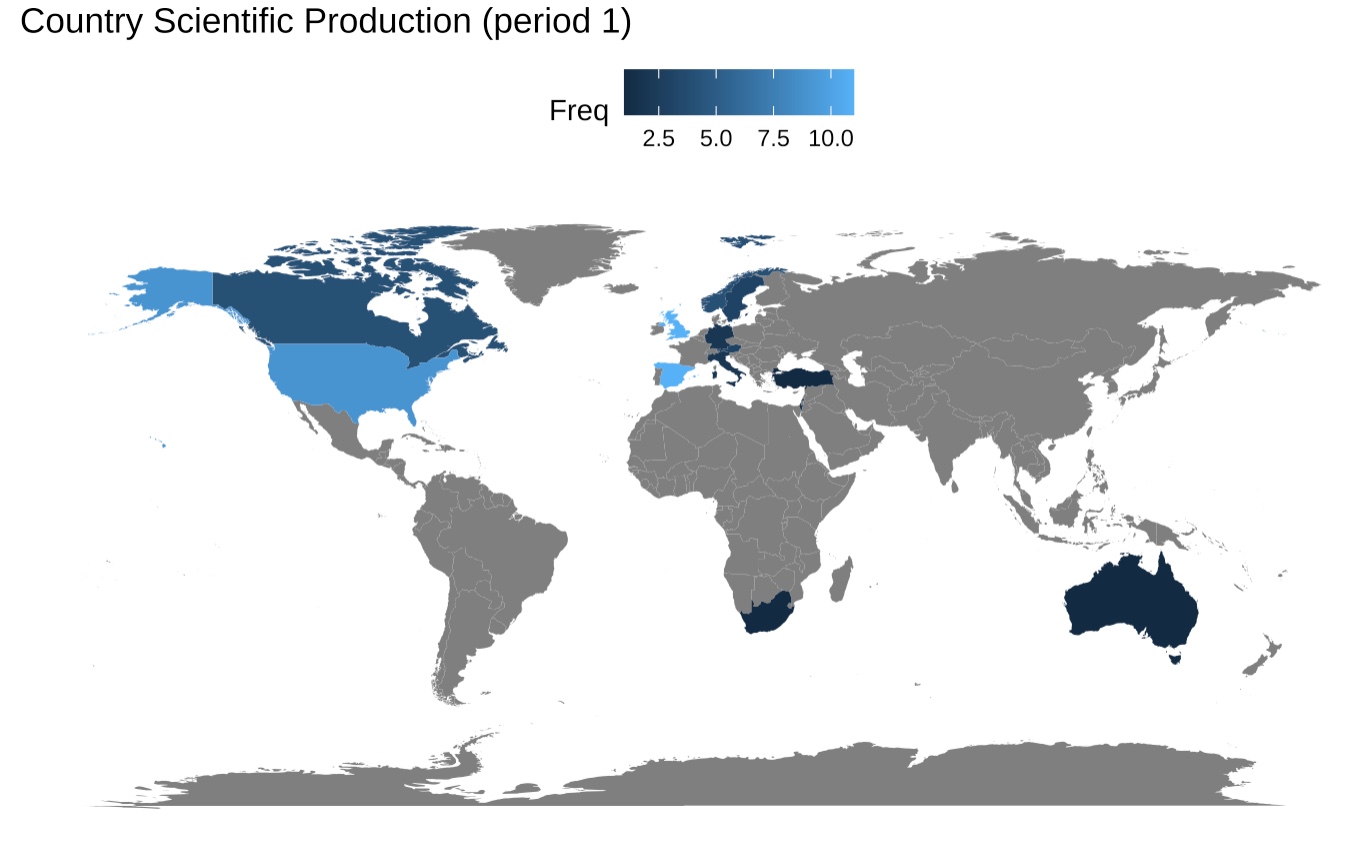


**Supplementary Figure 10.** Country’s Scientific Production (period 1).


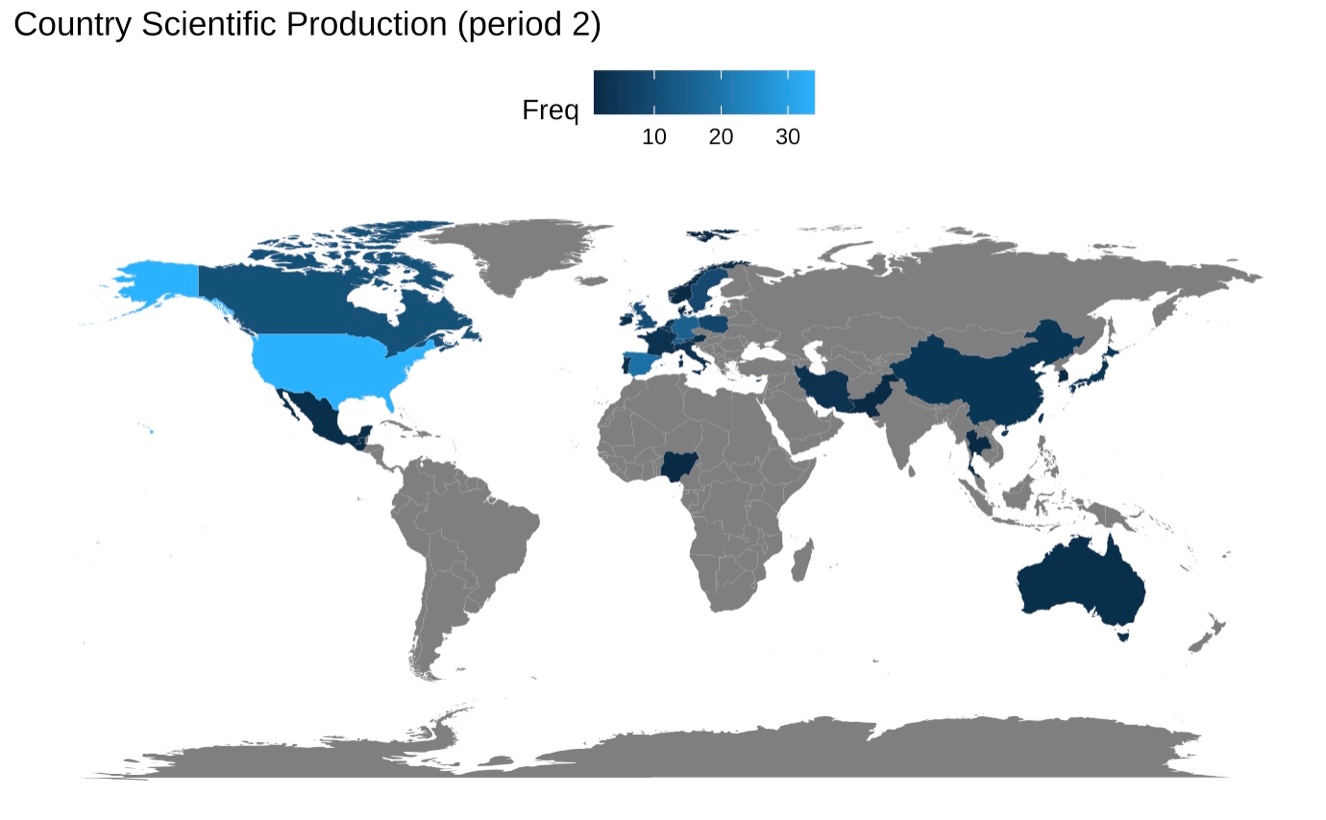


**Supplementary Figure 11.** Country’s Scientific Production (period 2).


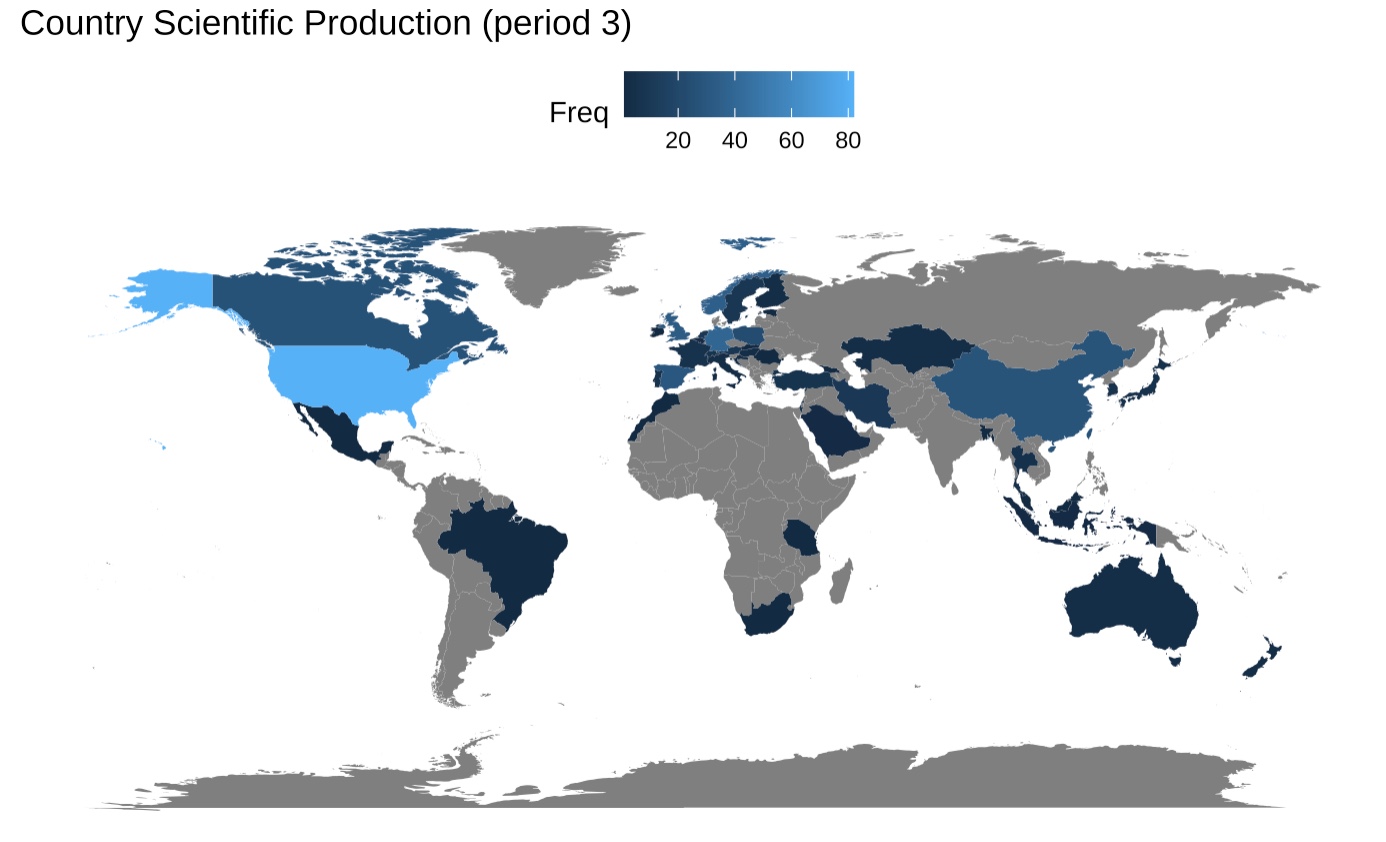


**Supplementary Figure 12.** Country’s Scientific Production (period 3).

**Supplementary Table 8.** Top ten most global cited documents in L3 acquisition in period 1.

| Ranking | Author(s) | Title | Year | Total Citations | TC per Year |
| --- | --- | --- | --- | --- | --- |
| 1 | Costa et al. | How do highly proficient bilinguals control their lexicalization process? Inhibitory and language-specific selection mechanisms are both functional | 2006 | 292 | 17.18 |
| 2 | Williams and Hammarberg | Language switches in L3 production: Implications for a Polyglot Speaking Model | 1998 | 220 | 8.80 |
| 3 | Cenoz, J. | The additive effect of bilingualism on third language acquisition: A review | 2003 | 217 | 10.85 |
| 4 | Dewaele et al. | Effects of trait emotional intelligence and sociobiographical variables on communicative anxiety and foreign language anxiety among adult multilinguals:  A review and empirical investigation | 2008 | 215 | 14.33 |
| 5 | Flynn et al. | The Cumulative-Enhancement Model for language acquisition: Comparing adults' and children's patterns of development in first, second and third language acquisition of relative clauses | 2004 | 212 | 11.16 |
| 6 | Bardel and Falk | The role of the second language in third language acquisition: The case of Germanic syntax | 2007 | 192 | 12.00 |
| 7 | Jessner, U. | Teaching third languages: Findings, trends and challenges | 2008 | 161 | 10.06 |
| 8 | Sanz, C. | Bilingual education enhances third language acquisition: Evidence from Catalonia | 2000 | 155 | 6.74 |
| 9 | Jessner, U. | Teaching third languages: Findings, trends and challenges | 2008 | 161 | 10.06 |
| 10 | Klein | Second versus third language acquisition: Is there a difference? | 1995 | 115 | 4.11 |

**Supplementary Table 9.** Top ten most global cited documents in L3 acquisition in period 2.

| Ranking | Author(s) | Title | Year | Total Citations | TC per Year |
| --- | --- | --- | --- | --- | --- |
| 1 | Rothman, J. | L3 syntactic transfer selectivity and typological determinacy: The Typological Primacy Model | 2011 | 168 | 14.00 |
| 2 | Cenoz, J. | The influence of bilingualism on third language acquisition: Focus on multilingualism | 2011 | 127 | 12.70 |
| 3 | Lüdi and Py | To be or not to be … a plurilingual speaker | 2009 | 110 | 7.86 |
| 4 | Rothman, J. | On the typological economy of syntactic transfer: Word order and relative clause high/low attachment preference in L3 Brazilian Portuguese | 2010 | 94 | 7.23 |
| 5 | Falk and Bardel | Object pronouns in German L3 syntax: Evidence for the L2 status factor | 2010 | 93 | 7.75 |
| 6 | Duff, P. | Identity, agency, and second language acquisition | 2013 | 91 | 9.10 |
| 7 | Rothman and Cabrelli | What variables condition syntactic transfer? A look at the L3 initial state | 2010 | 91 | 7.00 |
| 8 | Dewaele, J.-M | The link between foreign language classroom anxiety and psychoticism, extraversion, and neuroticism among adult bi- and multilinguals | 2013 | 80 | 8.00 |
| 9 | Falk and Bardel | The study of the role of the background languages  in third language acquisition. The state of the art | 2009 | 78 | 6.00 |
| 10 | Engel de Abreu, P. M. J., and Gathercole, S. E | Executive and phonological processes in second-language acquisition | 2012 | 76 | 6.91 |

**Supplementary Table 10.** Top ten most global cited documents in L3 acquisition in period 3.

| Ranking | Author(s) | Title | Year | Total Citations | TC per Year |
| --- | --- | --- | --- | --- | --- |
| 1 | Rothman, J. | Linguistic and cognitive motivations for the Typological Primacy Model (TPM) of third language (L3) transfer:  Timing of acquisition and proficiency considered | 2015 | 140 | 17.50 |
| 2 | Westergaard et al. | Crosslinguistic influence in the acquisition of a third language: The Linguistic Proximity Model | 2017 | 95 | 15.83 |
| 3 | Slabakova, R. | The scalpel model of third language acquisition | 2017 | 73 | 12.17 |
| 4 | Rothman et al. | Third language acquisition and linguistic transfer | 2019 | 46 | 11.50 |
| 5 | Ecke, P. | Parasitic vocabulary acquisition, cross-linguistic influence, and lexical retrieval in multilinguals | 2015 | 43 | 5.38 |
| 6 | Thompson and Erdil-Moody | Operationalizing multilingualism: Language learning motivation in Turkey | 2016 | 37 | 5.29 |
| 7 | González Alonso and Rothman | Coming of age in L3 initial stages transfer models: Deriving developmental predictions and looking towards the future | 2017 | 32 | 5.33 |
| 8 | Giancaspro et al. | Transfer at the initial stages of L3 Brazilian Portuguese: A look at three groups of English/Spanish bilinguals | 2015 | 32 | 4.00 |
| 9 | Puig-Mayenco et al. | A systematic review of transfer studies in third language acquisition | 2020 | 30 | 10.00 |
| 10 | Slabakova, R.  and Del Pilar García Mayo, M. | The L3 syntax–discourse interface | 2015 | 30 | 3.75 |

**Supplementary Table 11.** Collaboration Network (authors) by period.

| Period | Cluster | Node | Betweenness | Closeness | PageRank |
| --- | --- | --- | --- | --- | --- |
| 1 | 1 | Cenoz, J. | 0.00 | 1.00 | 0.5 |
|  |  | Valencia, Jf. | 0.00 | 1.00 | 0.5 |
| 2 | 1 | Bardel, C. | 1.00 | 0.50 | 0.12162162 |
|  |  | Lindqvist, C. | 0.00 | 0.33 | 0.06418919 |
|  |  | Falk, Y. | 0.00 | 0.33 | 0.06418919 |
|  | 2 | Bérubé, D. | 0.00 | 1.00 | 0.08333333 |
|  |  | Marinova-Todd, Sh. | 0.00 | 1.00 | 0.08333333 |
|  | 3 | Rothman, J. | 0.00 | 1.00 | 0.08333333 |
|  |  | Cabrelli, J. | 0.00 | 1.00 | 0.08333333 |
|  | 4 | Franceschini, R. | 0.00 | 0.25 | 0.08333333 |
|  |  | Luedi, G. | 0.00 | 0.25 | 0.08333333 |
|  |  | Nitsch, C. | 0.00 | 0.25 | 0.08333333 |
|  |  | Radue, Ew. | 0.00 | 0.25 | 0.08333333 |
|  |  | Zappatore, D. | 0.00 | 0.25 | 0.08333333 |
| 3 | 1 | Chen, Hc. | 0.00 | 1.00 | 0.03703704 |
|  |  | Han, Qw. | 0.00 | 1.00 | 0.03703704 |
|  | 2 | Lorenz, E. | 0.00 | 1.00 | 0.03703704 |
|  |  | Siemund, P. | 0.00 | 1.00 | 0.03703704 |
|  | 3 | Westergaard, M. | 0.00 | 1.00 | 0.03703704 |
|  |  | Mitrofanova, N. | 0.00 | 1.00 | 0.03703704 |
|  | 4 | Rothman, J. | 0.70 | 0.33 | 0.05430175 |
|  |  | Puig-Mayenco, E. | 0.00 | 0.25 | 0.0346579 |
|  |  | González Alonso, J. | 0.30 | 0.33 | 0.04192414 |
|  |  | Alemán Bañón, J. | 0.00 | 0.25 | 0.01726437 |
|  | 5 | Jabbari, A. | 0.00 | 1.00 | 0.03703704 |
|  |  | Fallah, N. | 0.00 | 1.00 | 0.03703704 |
|  | 6 | del Pilar García Mayo, M. | 0.00 | 1.00 | 0.03703704 |
|  |  | Slabakova, R. | 0.00 | 1.00 | 0.03703704 |
|  | 7 | Kupisch, T. | 0.00 | 0.50 | 0.0393864 |
|  |  | Lloyd-Smith, A. | 0.00 | 0.50 | 0.0393864 |
|  |  | Gyllstad, H. | 0.00 | 0.50 | 0.03233831 |
|  | 8 | Hulk, A. | 0.00 | 0.50 | 0.03703704 |
|  |  | Sleeman, P. | 0.00 | 0.50 | 0.03703704 |
|  |  | Stadt, R. | 0.00 | 0.50 | 0.03703704 |
|  | 9 | Wrembel, M. | 0.00 | 0.33 | 0.03953062 |
|  |  | Kopečková, R. | 0.00 | 0.33 | 0.03953062 |
|  |  | Balas, A. | 0.00 | 0.33 | 0.03454345 |
|  |  | Gut, U. | 0.00 | 0.33 | 0.03454345 |
|  | 10 | Bonilla, C. | 0.00 | 0.50 | 0.03703704 |
|  |  | Clark, M. | 0.00 | 0.50 | 0.03703704 |
|  |  | Golonka, E. | 0.00 | 0.50 | 0.03703704 |


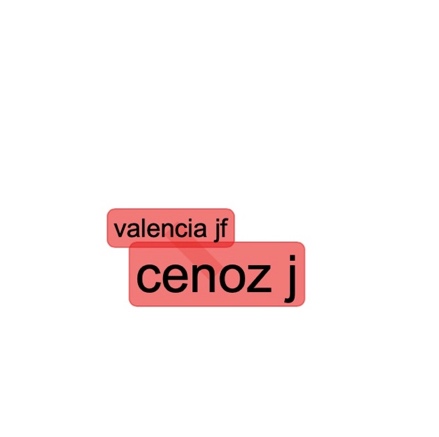


**Supplementary Figure 13.** Collaboration between authors (period 1).


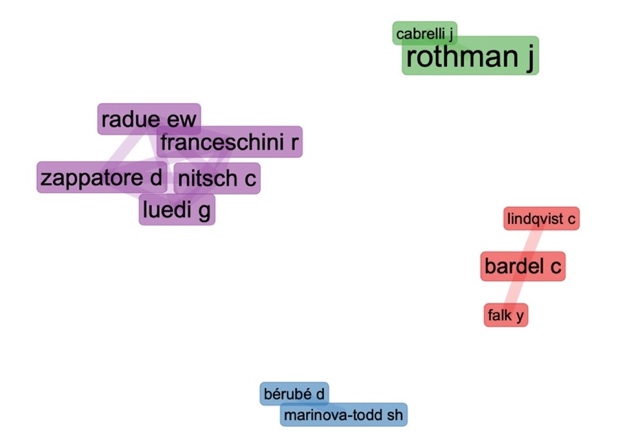


**Supplementary Figure 14.** Collaboration between authors (period 2).


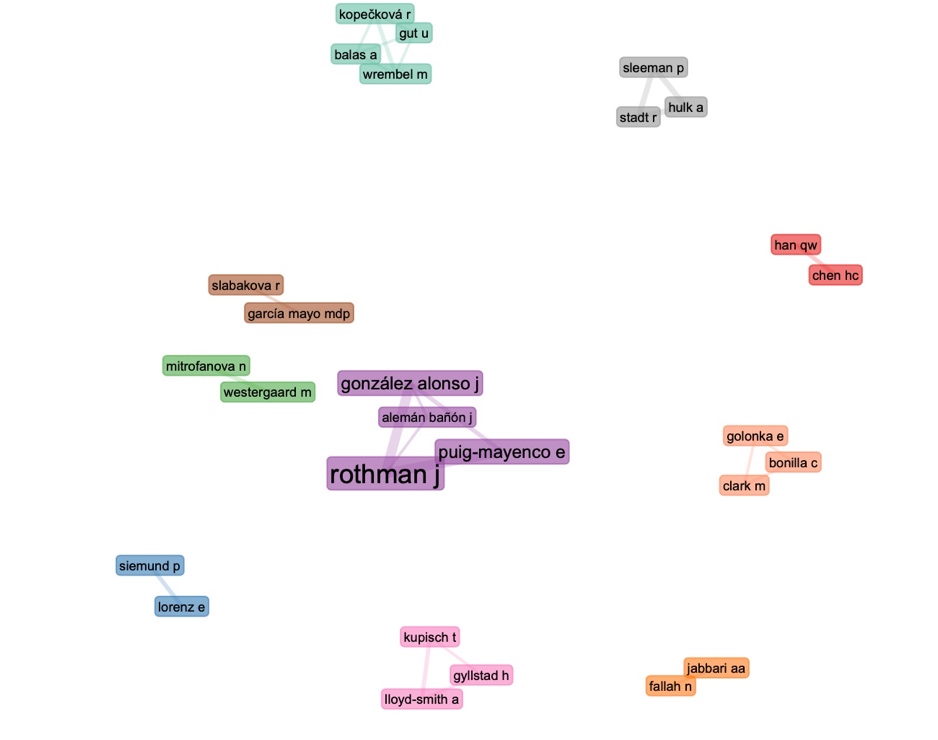


**Supplementary Figure 15.** Collaboration between authors (period 3).

**Supplementary Table 12.** Collaboration Network (affiliations) from 1984 to 2022.

| Cluster | Node | Country | Betweenness | Closeness | PageRank |
| --- | --- | --- | --- | --- | --- |
| 1 | UIT The Arctic University of Norway | Norway | 39.00 | 0.07 | 0.20 |
|  | University of Reading | UK | 0.00 | 0.04 | 0.05 |
|  | Norwegian University of Science And Technology | Norway | 9.00 | 0.05 | 0.07 |
|  | University of Konstanz | Germany | 0.00 | 0.05 | 0.05 |
|  | University of Hamburg | Germany | 0.00 | 0.03 | 0.02 |
|  | Lund University | Sweden | 0.00 | 0.05 | 0.04 |
| 2 | University of Illinois at Chicago | USA | 0.00 | 0.03 | 0.03 |
|  | King's College London | UK | 9.00 | 0.05 | 0.05 |
| 3 | University of the Basque Country | Spain | 0.00 | 0.04 | 0.03 |
|  | University of Southampton | UK | 17.00 | 0.05 | 0.08 |
|  | University of Iowa | USA | 0.00 | 0.04 | 0.03 |
| 4 | University of Basel | Switzerland | 0.00 | 1.00 | 0.06 |
|  | University Hospital of Basel | Switzerland | 0.00 | 1.00 | 0.06 |
| 5 | City University of Hong Kong | China | 0.00 | 1.00 | 0.06 |
|  | The Education University of Hong Kong | China | 0.00 | 1.00 | 0.06 |
| 6 | Adam Mickiewicz University | Poland | 0.00 | 1.00 | 0.06 |
|  | University of Münster | Germany | 0.00 | 1.00 | 0.06 |


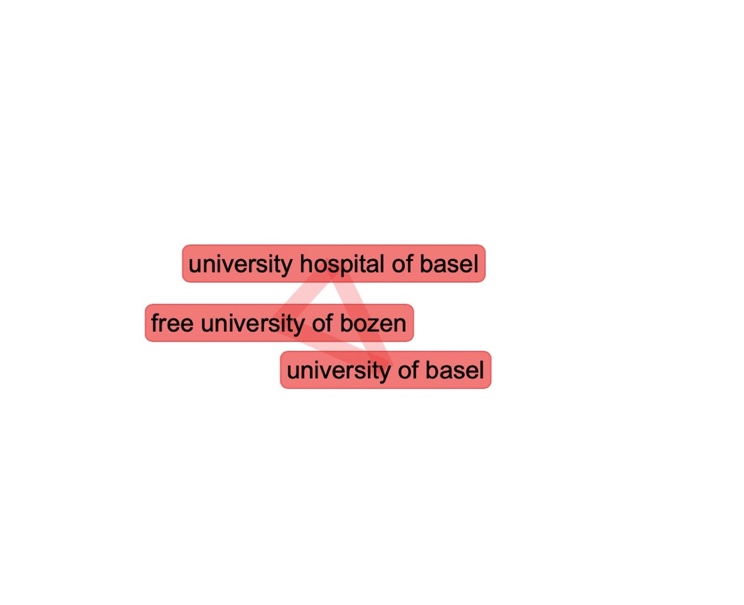


**Supplementary Figure 16.** Cluster plot of collaboration Network (affiliations) in period 2.


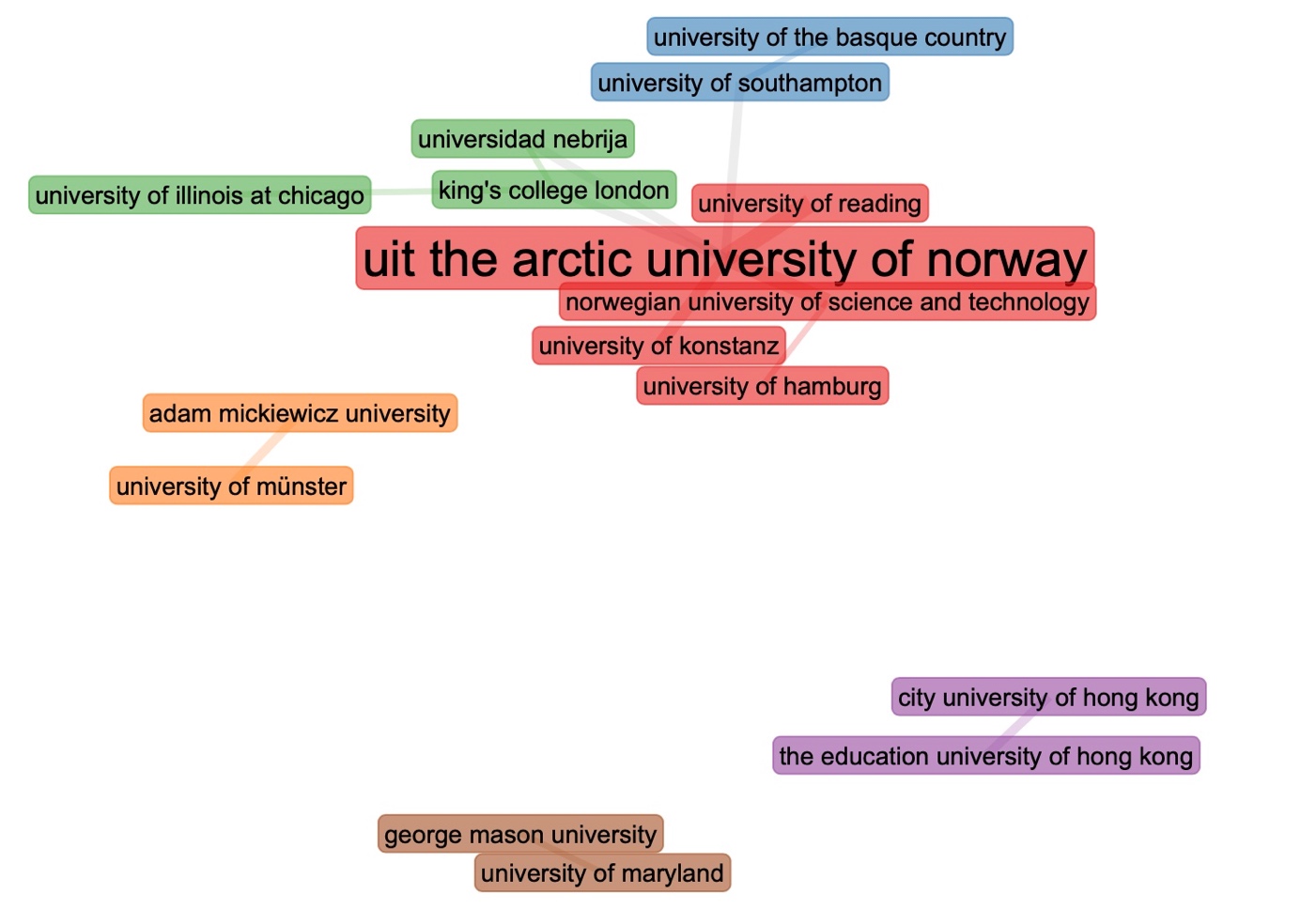


**Supplementary Figure 17.** Cluster plot of collaboration Network (affiliations) in period 3.

**Supplementary Table 13.** Collaboration Network (countries) by period.

| Period | Cluster | Node | Betweenness | Closeness | PageRank |
| --- | --- | --- | --- | --- | --- |
| 1 | 1 | USA | 0.00 | 1.00 | 0.50 |
|  |  | Canada | 0.00 | 1.00 | 0.50 |
| 2 | 1 | Germany | 0.00 | 0.33 | 0.11 |
|  |  | Switzerland | 1.00 | 0.50 | 0.21 |
|  |  | Italy | 0.00 | 0.33 | 0.11 |
|  | 2 | USA | 0.00 | 1.00 | 0.14 |
|  |  | Austria | 0.00 | 1.00 | 0.14 |
|  | 3 | Sweden | 0.00 | 1.00 | 0.14 |
|  |  | Netherlands | 0.00 | 1.00 | 0.14 |
| 3 | 1 | Germany | 17.95 | 0.10 | 0.16 |
|  |  | Poland | 0.00 | 0.06 | 0.03 |
|  |  | Netherlands | 0.00 | 0.06 | 0.05 |
|  |  | Sweden | 0.00 | 0.06 | 0.04 |
|  |  | Turkey | 0.00 | 0.06 | 0.02 |
|  | 2 | USA | 8.00 | 0.08 | 0.08 |
|  |  | Norway | 3.97 | 0.08 | 0.17 |
|  |  | UK | 0.00 | 0.07 | 0.13 |
|  |  | Spain | 1.08 | 0.08 | 0.12 |
|  | 3 | China | 0.00 | 0.05 | 0.02 |
|  | 4 | Austria | 0.00 | 1.00 | 0.08 |
|  | 5 | Hungary | 0.00 | 1.00 | 0.08 |


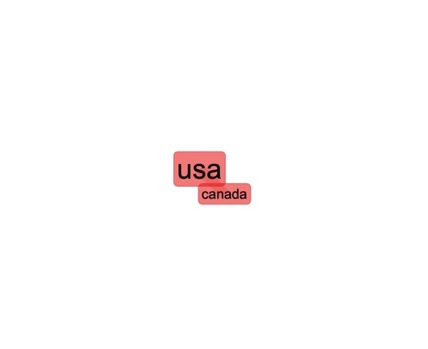


**Supplementary Figure 18.** Cluster plot of collaboration between authors of period 1.


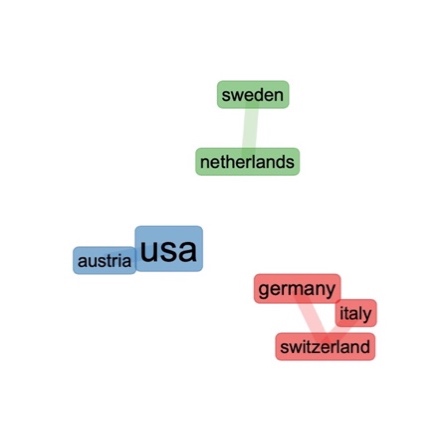


**Supplementary Figure 19.** Cluster plot of collaboration between authors of period 2.


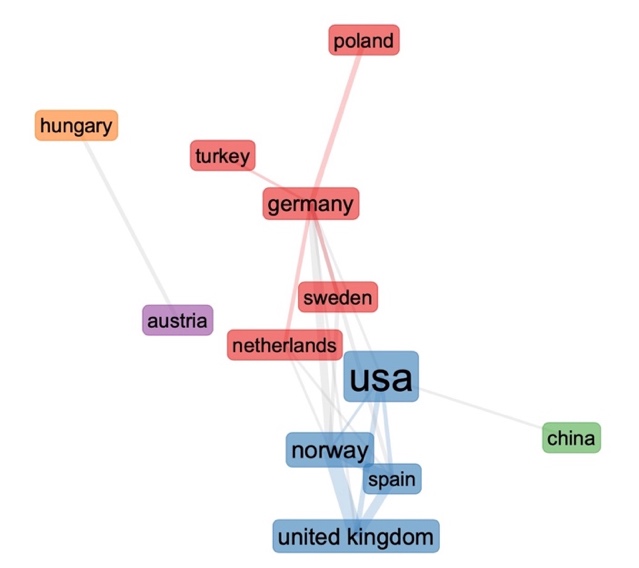


**Supplementary Figure 20.** Cluster plot of collaboration between authors of period 3.

**Supplementary Table 14.** Collaboration world map.

| **From** | **To** | **Frequency** |
| --- | --- | --- |
| Austria | Turkey | 1 |
| Austria | Netherlands | 1 |
| Austria | Hungary | 3 |
| Bangladesh | Malaysia | 1 |
| Canada | Sweden | 1 |
| Canada | Morocco | 1 |
| Canada | Israel | 1 |
| China | New Zealand | 1 |
| China | Malaysia | 1 |
| China | Japan | 1 |
| China | Bangladesh | 1 |
| France | Mexico | 1 |
| Germany | UK | 3 |
| Germany | Turkey | 2 |
| Germany | Switzerland | 2 |
| Germany | Sweden | 3 |
| Germany | Poland | 5 |
| Germany | Norway | 6 |
| Germany | Netherlands | 4 |
| Germany | Korea | 1 |
| Germany | Italy | 1 |
| Germany | France | 1 |
| Germany | Belgium | 1 |
| Iran | France | 1 |
| Korea | Singapore | 1 |
| Korea | Belgium | 1 |
| Netherlands | Korea | 1 |
| Netherlands | Estonia | 1 |
| Netherlands | Belgium | 1 |
| Norway | Turkey | 1 |
| Norway | Sweden | 3 |
| Norway | Netherlands | 2 |
| Singapore | Indonesia | 1 |
| Spain | UK | 7 |
| Spain | Sweden | 1 |
| Spain | Norway | 7 |
| Spain | Netherlands | 2 |
| Spain | Germany | 3 |
| Spain | Canada | 1 |
| Sweden | Netherlands | 3 |
| Switzerland | Italy | 2 |
| Turkey | Hungary | 1 |
| UK | Thailand | 1 |
| UK | Sweden | 1 |
| UK | Norway | 11 |
| UK | Netherlands | 1 |
| UK | Mexico | 1 |
| UK | Luxembourg | 1 |
| UK | Italy | 1 |
| UK | Ireland | 1 |
| UK | France | 1 |
| USA | UK | 5 |
| USA | Sweden | 1 |
| USA | Spain | 3 |
| USA | Poland | 1 |
| USA | Norway | 2 |
| USA | Netherlands | 2 |
| USA | Mexico | 1 |
| USA | Korea | 2 |
| USA | Japan | 1 |
| USA | Guatemala | 1 |
| USA | Germany | 3 |
| USA | Finland | 1 |
| USA | China | 3 |
| USA | Canada | 3 |
| USA | Belgium | 2 |
| USA | Austria | 3 |
| USA | Australia | 1 |
